# Engineering Ultrafast Molecular Rotors via Chalcogen bonds

**DOI:** 10.1021/jacs.6c02463

**Published:** 2026-04-27

**Authors:** Arun Dhaka, Antonio Macias Jr, Andrea Pizzi, Antonio Frontera, Riku Yamamoto, Stuart E. Brown, Miguel A. Garcia-Garibay, Giuseppe Resnati

**Affiliations:** † Laboratory of Nanostructured Fluorinated Materials (NFMLab), Department of Chemistry, Materials, and Chemical Engineering “Giulio Natta”, 18981 Politecnico di Milano, via E. Bassini 6, 20133 Milano, Italy; § Department of Chemistry and Biochemistry, 8783 University of California, Los Angeles, California 90095-1569, United States; ⊥ Department of Chemistry, Universitat de les Illes Balears, Crta. deValldemossa, Palma de Mallorca (Baleares) 07122, Spain; ∥ Department of Physics and Astronomy, University of California, Los Angeles, California 90095-1569, United States

## Abstract

Chalcogen bonds are emerging σ-hole interactions with untapped potential in amphidynamic materials. We report the first crystalline molecular rotors held by chalcogen bonds and their ultrafast rotational dynamics. The rotator component 1,4-diazabicyclo[2.2.2]­octane and phenylselenocyanate-based stators assemble via exceptionally short and highly directional Se···N contacts (Nc = 0.76–0.81; ∠NC–Se···N = 174–175 °). Solid-state ^1^H NMR T_1_ spin–lattice relaxation measurements reveal rotation at hundreds of MHz with low activation barriers (*E*_*a*_ = 1.22–2.78 kcal mol^–1^), in agreement with the packing coefficient and computational analysis. These findings highlight chalcogen bonds as a powerful tool for designing robust, crystalline molecular machines.

## Graphical abstract

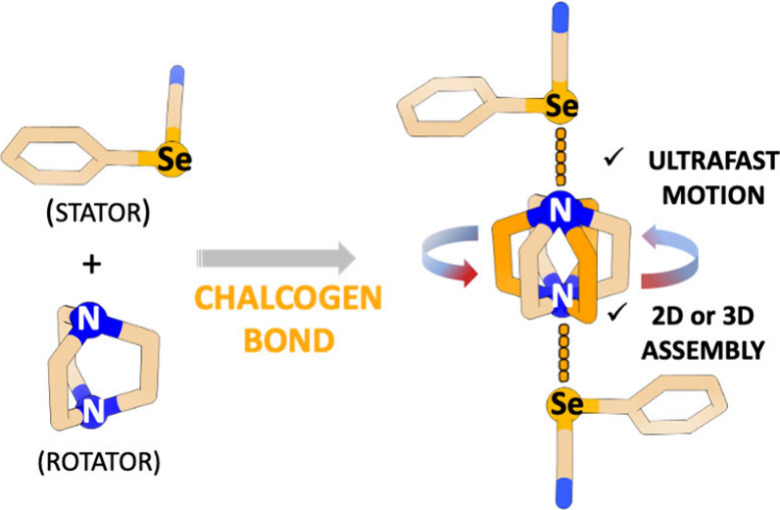

Biomolecular machines are dynamic structures that drive essential enzymatic activities through precise molecular motions.^1^ This has inspired the development of artificial molecular machines, where motion is structurally programmed at the molecular level to perform specific mechanical actions in response to external stimuli e.g., rotaxane and catenane based molecular motors, logic gates, shuttles, switches etc.^2^ Among the various modes of motion, molecular rotation with low energy barriers can be engineered in the lattice of organic crystals to achieve molecular rotors or gyroscopes.^3^ For instance, phenylene and bicyclo[2.2.2]­octane groups can undergo fast, thermally activated rotation while being pinned along a single rotational axis, either covalently or noncovalently, within a static solid-state framework.^4^ Such amphidynamic materials combine static (stator) and dynamic (rotator) components and represent an emerging class of condensed matter with potential for switchable dielectrics,^5^ soft robotics, and artificial molecular machines.^6^ Crystal engineering offers strategies for the construction of molecular rotors by organizing stators and rotators into cocrystals, where the use of diverse supramolecular synthons ensures efficient assembly and allows fine-tuning of rotor dynamics through control of noncovalent interactions.^7^ Apart from hydrogen bond^8^ and metal coordination,^9^ σ-hole interactions like halogen bond (HaB) have been implemented in design of 1,4-diazabicyclo[2.2.2]­octane (DABCO) based molecular rotors, thanks to the directional nature of HaB (Scheme 1a).^10^ In the σ-hole category, chalcogen bond (ChB) is yet another excellent emerging tool that finds applications in the field of crystal engineering,^11^ catalysis,^12^ anion recognition^13^ and molecular balances,^14a^ actuators^14b^ etc. However, to the best of our knowledge, ChB has never been used in building molecular rotors. In this work, we report the first examples of crystalline ultrafast molecular rotors assembled under short and directional Se···N ChBs between phenyl-selenocyante-based ChB donor and DABCO as ChB acceptor (Scheme 1a).

**Scheme 1:**
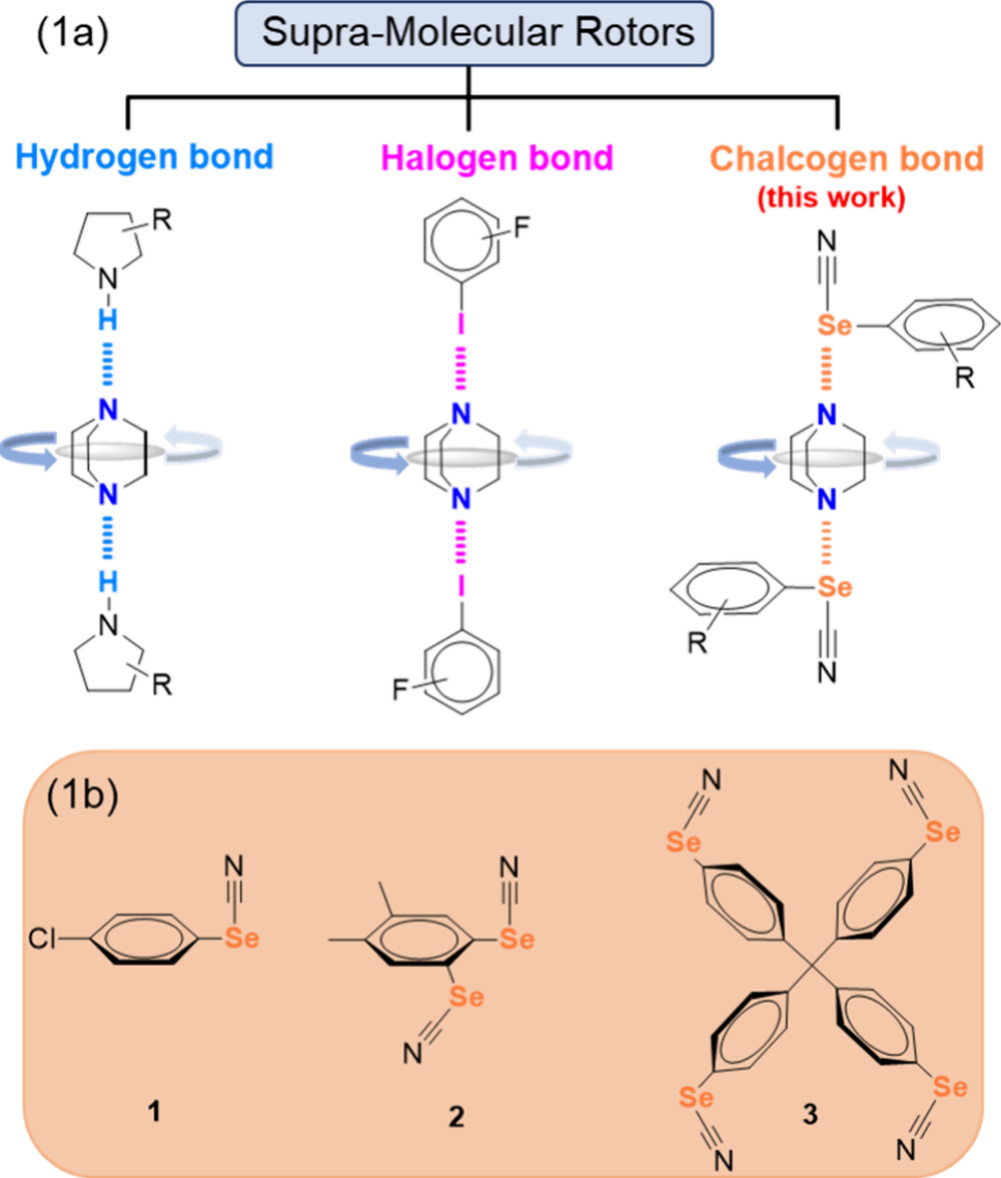
(a) Representation of Supramolecular Rotors Based on Hydrogen, Halogen and Chalcogen Bond Featuring DABCO as a Rotator; (b) Molecular Structures of Selenocyanate Based ChB Donors Investigated in This Study

ChB arises from electron-deficient σ-holes on covalently bonded chalcogens (R–Ch), which form non-covalent Ch•••Nu interactions with nucleophiles.^15^ Neutral donors such as R–SeCN^16^ and R–C≡C–(Se)­Me^17^ generate prominent σ-holes, enabling short and predictable interactions.^18^ In our design, we selected phenyl–SeCN derivatives as ChB donors anticipating that the rigid phenyl ring would serve as a static platform while chalcogen bonding would selectively pin DABCO along the N≡C–Se axis to define a reference axis of rotation (Scheme 1a, right).

To explore this, we synthesized a series of mono (**1**)-, bi (**2**)- and tetradentate (**3**) ChB donors (Scheme 1b) via selenocyanation of arylboronic esters with SeO_2_ and malonitrile (see the Supporting Information (SI)).^19^ Self-assembly of these donors with DABCO in ethyl acetate yielded single crystals suitable for X-ray diffraction. In each case, the crystal packing revealed that DABCO is uniaxially anchored between two N≡C–Se moieties *via* short and linear Se···N ChBs as anticipated, establishing a robust platform for stator-rotator organization (Figure 1, Table S1).

**Figure 1:**
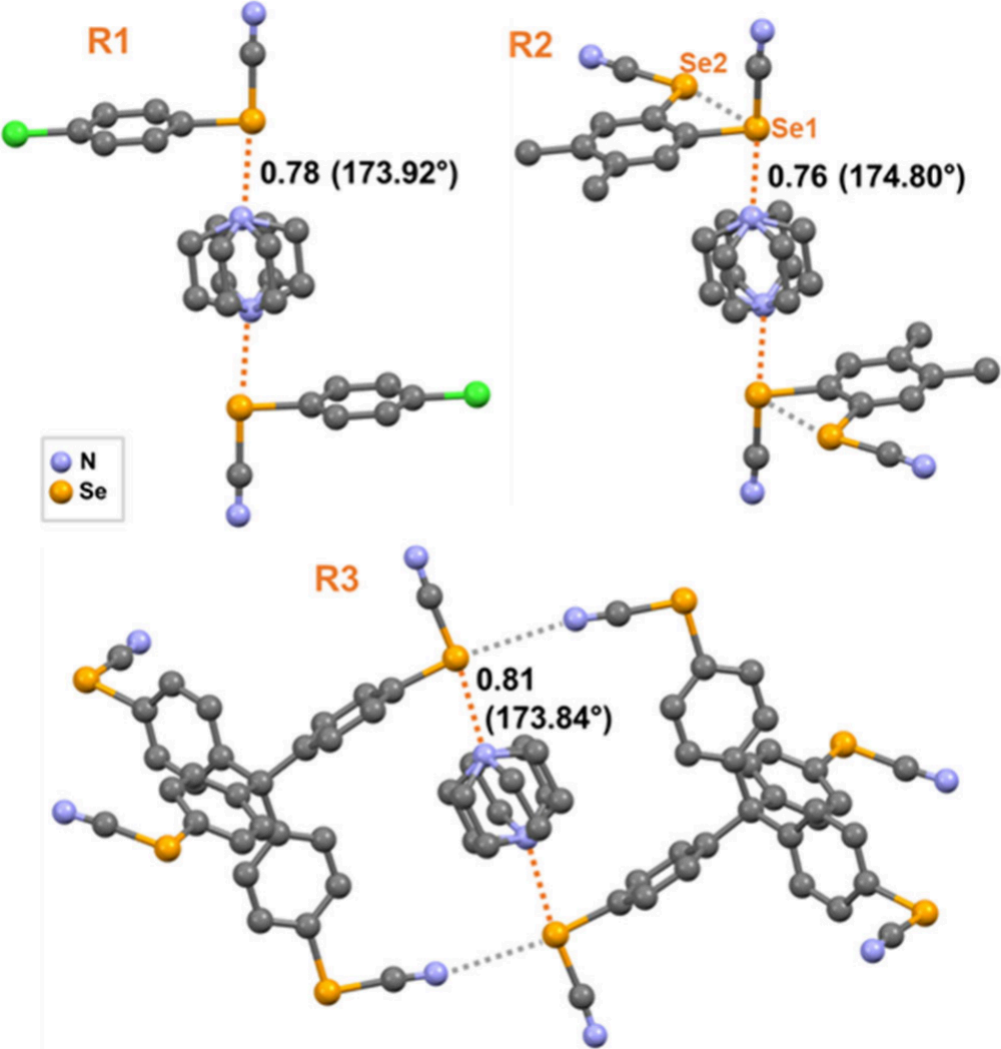
Crystal structures of **R1**–**R3** showing DABCO pinning via Se···N contacts. **R2** features an intramolecular Se2···Se1 ChB (3.217 Å; 158.5°). **R3** shows a Ph–Se···N≡C ChB involving the C–Se σ-hole (Nc = 0.97; 170.6°). Hydrogens are omitted.

For instance, cocrystal **R1** (donor **1** + DABCO) crystallizes in monoclinic system (SG *P*2_1_/*c*), forming the desired trimeric motif stabilized by very short N≡C–Se···N­(dabco) contacts (Figure 1, top left). The Se···N­(dabco) distance of 2.723 Å corresponds to a normalized contact^20,21^ (Nc) of 0.78 and exhibits excellent directionality with ∠NC–Se1···N = 173.9°. These trimer motifs are isolated from each other without further ChB interaction (Figure S1).

A bidentate ChB donor (**2**), bearing 3,4-substituted methyl groups, was selected to further probe the ability of SeCN in delivering a reliable stator-rotator framework. The resulting cocrystal **R2** (donor **2** + DABCO) similarly pins DABCO (Figure 1, top right) with even shorter and more linear Se···N­(dabco) ChBs [Nc = 0.76, ∠NC–Se1···N = 174.8°]. The donor **2** contains two SeCN groups, one engaging in intermolecular ChB with DABCO and the other conformationally locked via an intramolecular Se2···Se1 ChB where the σ-hole along the N≡C–Se2 bond interacts attractively with one lone pair on Se1. Similar intramolecular Se···Se ChBs are known in ortho-substituted Ph-(SeCN)_2_ derivatives^22^ (Figure S2), conferring conformational rigidity that persists in the DABCO cocrystal **R2**. These ChB bonded trimeric units are extended into a 2D sheet type network through the recurrent antiparallel pairing of Se–C≡N units (Figure S3).^22^ This involves the second σ-hole on selenium atoms present along the phenyl–Se bond and nitrogen lone pairs of the CN group (Figure S4). Overall, the assembly process is quite systematic in combining the σ-hole along N≡C–Se bond with stronger Lewis-bases (N of dabco) and the σ-hole along Ph–Se bond with weaker Lewis-bases (N of CN).

To probe the generality of this approach, a tetradentate ChB donor (**3**) based on a tetraphenylmethane scaffold was designed. In cocrystal **R3** (**3** + DABCO), Se···N contacts pin DABCO within a cavity, an arrangement ideally suited for a rotator component (Figure 1, bottom). These dimers align orthogonally and propagate into 1D chains. The chains further connect through antiparallel SeCN pairing to give a porous 3D ChB network (Figure S5), which interpenetrates with two identical orthogonal nets to afford a 2-fold interpenetrated structure (Figure S6). These structures represent the first example of neutral ChB based cocrystals spanning 0D to 3D architectures as a function of donor denticity, advancing supramolecular design principles.^23^

Each donor produced a homogeneous cocrystal, as confirmed by powder X-ray diffraction (Figure S7). In all structures, the DABCO units display persistent rotational disorder along the N–N axis, even upon cooling to 100 K (Figure 2 and Figure S8), suggesting that this dynamic motion is an intrinsic feature of the lattice rather than a purely thermal effect. Subtle changes in the thermal ellipsoids at low temperatures indicate a redistribution of dynamic modes rather than a freezing of rotation. Consistent with this, differential scanning calorimetry revealed no phase transitions upon cooling (Figure S9), highlighting the stability and resilience of these cocrystal frameworks.

**Figure 2:**
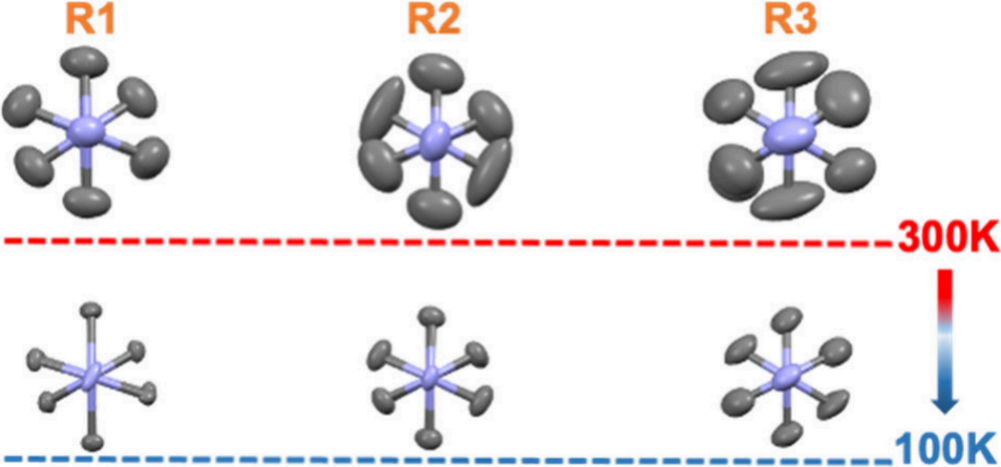
Views along the N–N axis of DABCO in cocrystals **R1**, **R2**, and **R3** at RT and 100 K. DABCO is split into two positions with an occupancy of around 50%.

^1^H–^13^C cross-polarization magic angle spinning (CPMAS)^24^ solid-state NMR spectra of the three rotors was measured, and the acquired spectra are consistent with their crystal structures showing a sharp DABCO peak and the expected number of signals (Figures S10–S12). Moreover, the signal-to-noise ratio in the spectra is consistent with the relaxation time constant (T_1_) values obtained at room temperature (Figure S13–S15). A hydrogen nucleus with a shorter T_1_ can more quickly build magnetization, leading to a better signal-to-noise ratio. To analyze the cocrystal’s rotational dynamics, we leveraged solid-state ^1^H NMR T_1_ spin–lattice relaxation measurements. This technique is sensitive to magnetic field fluctuations near the Larmor frequency (ω_0_) of the nuclei of interest, which are caused primarily by the motion-induced modulation of homonuclear dipolar coupling.^8,10,25–28^ Therefore, systems containing rotating elements bearing the probed magnetic nuclei will experience a decrease in their T_1_ values. The relaxation rate of the crystalline systems can be described by the Kubo-Tomita equation (eq 1)­

1 $$\frac{1}{T_{1}}=C\left(\frac{\tau_{c}}{1+{\left(\omega_{0}\tau_{c}\right)}^{2}}+\frac{4\tau_{c}}{1+4{\left(\omega_{0}\tau_{c}\right)}^{2}}\right)$$

where C describes the strength of dipolar coupling between probed nuclei and *τ*_*c*_ is the correlation time for rotary motion.^28^ Furthermore, if the mechanism facilitating relaxation is thermally activated, the activation parameters (*E*_*a*_, Δ*H* ^‡^ and Δ*S* ^‡^) for rotation can be determined by analyzing the temperature-dependence of *τ*_*c*_ through an Arrhenius (eq S2) or Eyring (eq S3) relation (see SI).^26^

The activation parameters determined from fitting the temperature-dependence of the rotors’ relaxation rate to the Kubo-Tomita equation (Figure 3 and Figures S16–S18) are presented in Table 1. We found that the three rotors have comparable dipolar coupling constant values (C = (1.7–3.3) × 10^9^ s^–2^), as expected for molecular rotors with the same number of hydrogen nuclei facilitating magnetic relaxation, with differences observed potentially attributed to intermolecular contributions to dipolar coupling.^29^ Activation energy (*E*_*a*_) observed for **R1** (2.78 kcal/mol) is significantly greater from those of **R2** and **R3** (1.22 and 1.43 kcal/mol, respectively). Notably, these *E*_*a*_ values are much lower than those observed for analogous HaB based molecular rotors (*E*_*a*_ = 2.4–4.9 kcal/mol)^10^ i.e., cocrystals of fluorinated iodobenzenes and DABCO in Scheme 1. Moreover, while the reported HaB systems are restricted to discrete 0D assemblies, the present systems form extended networks thanks to the dual σ-hole character of chalcogen atoms. Steric effects play a crucial role in rotary behavior and can explain the determined *E*_*a*_ values.^26,29^ **R1** exhibits the largest rotational activation energy (*E*_*a*_ = 2.78 kcal mol^–1^), suggesting that the lack of extended ChB may lead to a denser crystal structure.

**Table 1:** Activation Parameters for Rotational Dynamics of **R1**, **R2** and **R3** from Kubo-Tomita Fit of T_1_ Data

	*E*_*a*_ (kcal mol^–1^)	τ_0_^–1^ (10^12^ s^–1^)	Δ*H* ^‡^ (kcal mol^–1^)	Δ*S* ^‡^ (cal mol^–1^ K^–1^)
**R1**	2.78 ± 0.05	5.28 ± 0.05	1.18 ± 0.03	–14.6 ± 0.04
**R2**	1.22 ± 0.05	2.17 ± 0.06	1.03 ± 0.07	–1.77 ± 0.07
**R3**	1.43 ± 0.03	0.49 ± 0.01	1.09 ± 0.03	–6.05 ± 0.03

**Figure 3:**
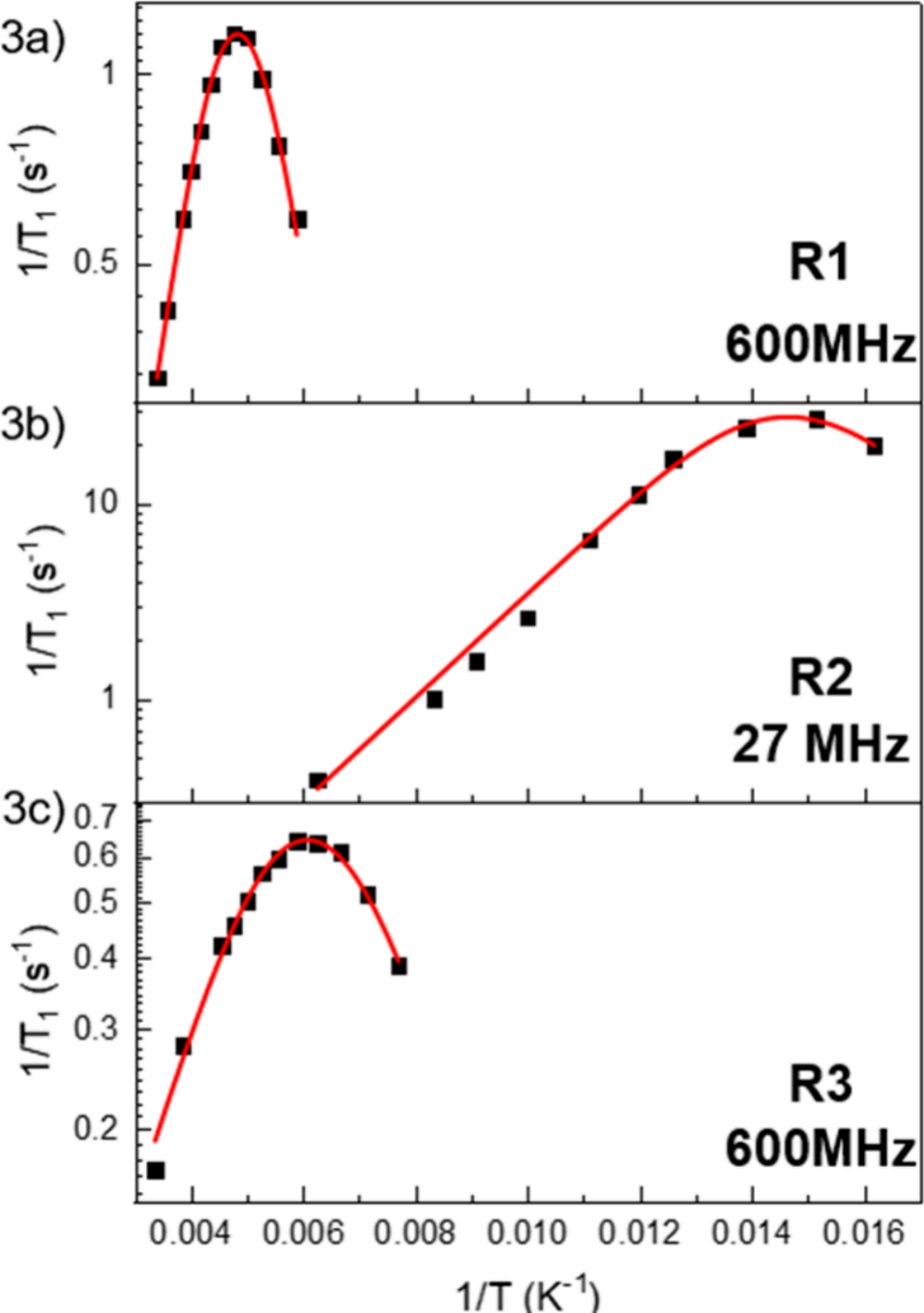
Kubo-Tomita fitting (red line) of experimentally measured spin–lattice relaxation rates (black squares) for (a) **R1**, (b) **R2**, and (c) **R3** with their respective Larmor frequencies.

Eyring analysis reveals a similar activation enthalpy in contrast to significant differences in activation entropy contributions. As shown in Table 1, complex **R1** has a large negative entropy of activation (Δ*S* ^‡^ = −14.59 kcal mol^–1^ K^–1^), suggesting that it has the greatest change in going from its rotational ground state to its transition state, as compared to rotors **R2** and **R3**. If we consider positional disorder and assume all rotors have a single well-defined transition state and therefore the same entropy, then we might expect a smaller disorder from **R1** in the ground state, which is consistent with the smaller thermal ellipsoids (Figure 2).

To further rationalize the different rotational behaviors observed, the packing coefficient of DABCO was calculated for **R1**, **R2**, and **R3** systems as a measure of space-filling efficiency within the rotator cavity. The results are 56, 52, and 50% for **R1**, **R2**, and **R3**, respectively (see SI). The larger value for **R1** (>55%) indicates a more congested environment, which is in good agreement with its higher activation energy and more negative activation entropy. In the case of **R2** and **R3**, the lower and more similar packing coefficients are consistent with their lower activation energies (see Table 1), even though the exact numerical trend is not perfectly reproduced.

All three rotors show a single maximum in Figure 3, which implies a single mechanism for relaxation. This maximum results from rotation at the Larmor frequency, so it is worth noting this frequency matching occurs at different temperatures for the rotors.^28^ The temperature at which this happens reflects the rotational activation energy, with larger energy barriers requiring higher temperatures to rotate at the Larmor frequency. Furthermore, the size of the energy barrier also influences the shape of the parabolic curve, with larger barriers giving steeper curves and vice versa.

Density functional theory (DFT) calculations were performed to estimate the rotational barriers and support the experimental observations (see SI for details). The calculated activation energies (*E*_a_) for **R1**, **R2**, and **R3** are 2.2, 1.0, and 1.5 kcal mol^–1^, respectively, showing excellent agreement with the experimental barriers of 2.78, 1.22, and 1.43 kcal mol^–1^ (Figure 4). The near identical energies of the 60° and 0° rotamers align with the ∼50% occupancy ratio observed in the X-ray structures. Analysis of the energy components at the transition states reveals that exchange repulsion (E_ex‑rep_) is the primary contributor to the rotational barrier. The largest E_ex‑rep_ contribution is observed in **R1** (6.0 kcal mol^–1^), which is consistent with its higher activation energy and larger packing coefficient. In contrast, the dispersion (E_disp_) contributions showed the smallest variation across the series. These results confirm that the rotational dynamics are primarily governed by steric interactions within the chalcogen-bonded framework.

**Figure 4:**
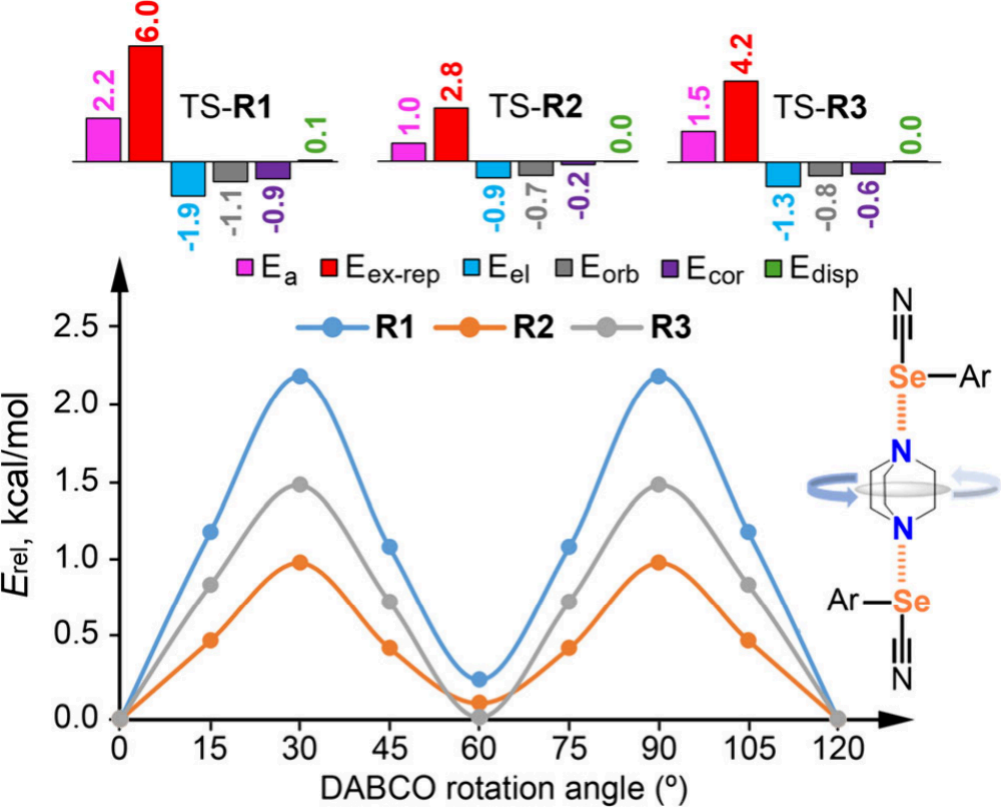
(top) Calculated activation energy (*E*_a_, fuchsia) and individual energy contributions (kcal mol^–1^) to *E*_*a*_ at the transition states (TS). (bottom) Relative energy (E_rel_) vs DABCO rotation angle (0–120°).

In summary, chalcogen-bonded molecular rotors were constructed using DABCO as the rotator and a series of selenocyanate-based stators with varying ChB denticities (mono-, bi-, tetradentate). In all cases, crystal structures showed that DABCO was uniaxially anchored between two N≡C–Se moieties via short, nearly linear Se···N ChBs, providing a robust platform for supramolecular rotors. Variation in stator denticity directed assemblies from 0D to 2D sheets and 3D interpenetrated networks. Solid-state T_1_ spin–lattice relaxation measurements showed low rotational barriers (1.22–2.78 kcal mol^–1^), enabling ultrafast rotation at room temperature. DFT calculated barriers show excellent agreement with experimental values. Along with packing coefficients of 50–56%, the EDA results confirm that E_ex‑rep_ is the primary origin of the rotational barriers. These findings advance the design of crystalline molecular machines and amphidynamic materials with 2D or 3D architectures through robust chalcogen bonding.

## Supplementary Material





## Notes

Cartesian coordinates used in computational analysis (PDF)
Experimental procedures and NMR and crystallographic characterizations (PDF)
