# Supplementary material for: Engineering Ultrafast Molecular Rotors via Chalcogen bonds
Source: J Am Chem Soc. 2026 Apr 27;148(18):18591–6. doi: 10.1021/jacs.6c02463 (PMC13184984; doi:10.1021/jacs.6c02463)
Supplement: Supplementary file 1 [file ja6c02463_si_001.pdf]

SUPPORTING INFORMATION

Engineering Ultrafast Molecular Rotors via Chalcogen Bonds

Arun Dhaka,[†*] Antonio Macias Jr,[§] Andrea Pizzi,[†*] Antonio Frontera,[⊥] Riku Yamamoto,[||] Stuart E. Brown,[||] Miguel A. Garcia-Garibay,[§*] Giuseppe Resnati,[†]

[†]Laboratory of Nanostructured Fluorinated Materials (NFMLab), Department of Chemistry, Materials, and Chemical Engineering "Giulio Natta", Politecnico di Milano, via E. Bassini 6, 20133, Milano, Italy. [§]Department of Chemistry and Biochemistry, University of California, Los Angeles, California 90095-1569, United States. [⊥]Department of Chemistry, Universitat de les Illes Balears, Crta. deValldemossa, Palma de Mallorca (Baleares) 07122, Spain. [||]Department of Physics and Astronomy, University of California, Los Angeles, California 90095-1569, United States.

TABLE OF CONTENT

General Considerations……….……………………………………….………………....**page 3**

Synthesis ………………………………………………………………........…....**page 4**

Co-Crystal characterization and analysis………………………………..………...**page 6-16**

(a) Co-crystallization experiments

(b) Co-crystal structure details

(c) P-xrd data

(d) DSC data

Solid-state NMR studies………..........................……………..………..………...**page 17-23**

(a) ^{13}C CPMAS ssNMR

(b) T1 spin relaxation data

(c) Kubo-tomita Fitting of T1 data

Computational studies of rotational dynamics…………………....……………...**page 24-25**

(a) Packing coefficient calculations

(b) Rotational barrier calculations

References…………………………………...…………………………...…..…..**page 26**

1. General Considerations:

Selenium dioxide, malonitrile, aryl-pinacolato boronates, anhydrous dimethylsulfoxide, dioxane, ethyl acetate, chloroform were purchased from commercial suppliers (abcr and Sigma-Aldrich) and used without further purification.

^{1}H-NMR spectra were recorded at ambient temperature on Nuclear Magnetic Resonance Spectrometer AVANCE III, Bruker-BioSpin. All the chemical shifts are given in ppm and the Js in Hz. DMSO-d_6 was used as both solvent and internal standard in NMR spectra.

DSC analyses were performed with a Mettler Toledo DSC823e instrument, using aluminum light 20μL sample pans and Mettler STARe software for calculations.

PXRD measurements were performed with a Bruker AXS D8 powder diffractometer, with experimental parameters as follows: Cu-Kα radiation (λ = 1.54056 Å), scanning interval 5-40° at 2θ, step size 0.009°, exposure time 1.5 s per step. Simulations were generated using Mercury 4.2.0 software from the Crystallographic Data Center. Comparisons between experimental PXRD patterns and simulated patterns from the single-crystal structure were used to verify sample phase purity.

The single crystal data were collected using a XtaLAB Synergy diffractometer, equipped with a HyPix detector. Unit cell refinement and data reduction were performed using CrysAlisPro 1.171.41.98a. Structures were solved by direct methods using SHELXT and refined by full-matrix least-squares on F^2 with anisotropic displacement parameters for the non-H atoms using Olex2. Absorption correction was performed based on multi-scan procedure.

Solid-state NMR (ssNMR) measurements were recorded either on a Bruker Avance 600 MHz NMR spectrometer at 600 MHz (^{1}H) and 151 MHz (^{13}C) or at 27 MHz (^{1}H). Spectra were collected using natural abundance isotopes.

T_1 relaxation measurements were carried out using a saturation-recovery pulse sequence with varying *t* between 90^{0} pulses. 50mg of crystalline sample were packed into a 4cm long NMR tube cut from a regular 5mm Pyrex NMR tube. The vessel was capped with Teflon tape on both ends. T_1 values were obtained from exponential fitting the signal integral as a function of *t* according to:

$$A(t) = A_0\left(1 - e^{-\frac{t}{T_1}}\right). \quad (Eq.S1)$$

^{13}C cross-polarization magic angle spinning (CPMAS) ssNMR spectra were collected using 30-50mg of crystalline sample tightly packed in a zirconia rotor. Samples were spun at 18 kHz,

and spectra were recorded with a contact time of 2.5s and a recycle delay time of 5s with 256 scans at ambient temperature.

The activation parameters ($\Delta H^{\ddagger}$ and $\Delta S^{\ddagger}$) for rotation can be determined by analyzing the temperature-dependence of τ_c through an Arrhenius (Eq. S2) or Eyring (Eq. S3) relation

$$\tau_c = \tau_0 exp\left(\frac{E_a}{RT}\right) \quad (Eq. S2)$$

$$\tau_c = \left(\frac{h}{k_B T}\right) exp\left(\frac{\Delta H^{\ddagger}}{RT}\right) exp\left(\frac{-\Delta S^{\ddagger}}{R}\right) \quad (Eq. S3)$$

where, τ_0 can be seen as the inverse of the attempt frequency, E_a is the energy of activation for rotation, h is Planck's constant, k_B is Boltzmann's constant, R is the natural gas constant, $\Delta H^{\ddagger}$ is the activation enthalpy for rotation, and $\Delta S^{\ddagger}$ is the activation entropy for rotation.

2. Synthesis:

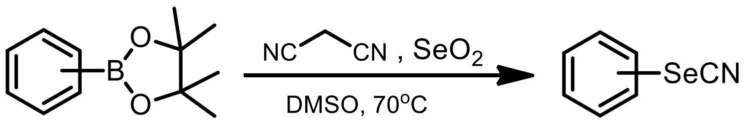

General procedure for aryl-SeCN:[1]

Malontrile (X eq) and Selenium oxide (3X eq) were placed in a 50ml over dried round bottom and anhydrous DMSO (5ml) was added. The reaction mixture was stirred for 30 min and until a dark orange color along with evolution of a gas was observed. Tetra-Bpin(X=4)-Phenylmethane (1 eq) was added and reaction mixture was heated at 70°C overnight. Flask was cooled to room temperature and water 10ml was added. Aqueous layer was washed with EtOAc (3 times) and combined organic layers were evaporated under reduced pressure using rotary evaporator. The crude solid residue was subjected to flash column chromatography on silica gel for purification (eluent: petroleum ether/ethyl acetate) to afford the desired aryl-SeCN compounds.

Compounds **1** and **2** are known and were confirmed by ^{1}H-NMR data. Compound **3** is a new tetradentate chalcogen bond donor and was structurally characterized by NMR and single P-xrd.

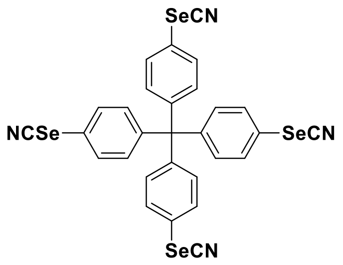

Compound 3: Yield: 38%. white solid. ^{1}H NMR (400 MHz, DMSO-d$_6$): δ 7.69 (d, J = 8.6 Hz, 8H), 7.32 (d, J = 8.7 Hz, 8H). ^{13}C NMR (101 MHz, DMSO-d$_6$): δ 64.58, 105.58, 122.58, 132.17, 133.86, 147.10.

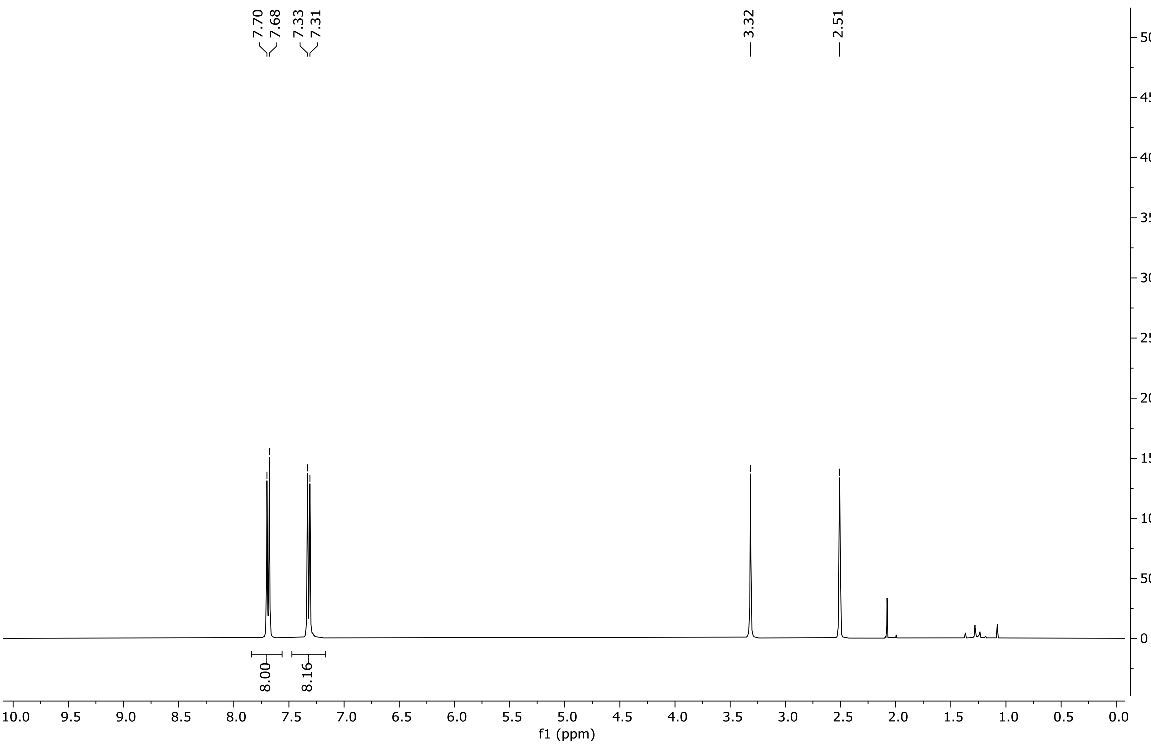

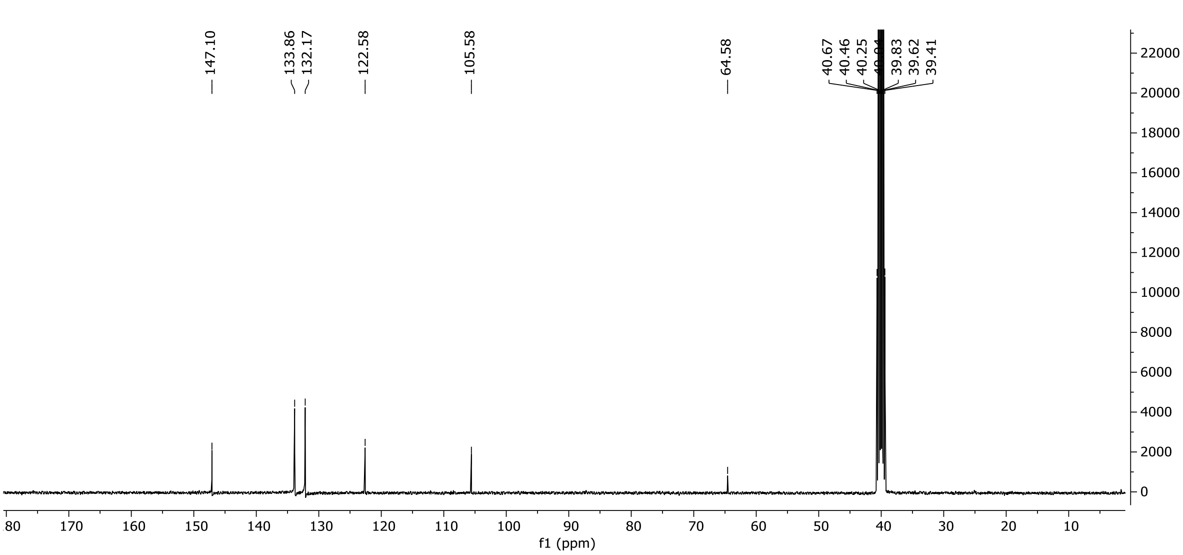

3. Co-Crystal characterization and analysis:

(a) Co-crystallization Experiments:

Experiments were performed in reference to ChB donors, on a scale of 50-100 mg.

R1: Slow evaporation of an ethylacetate solution containing compound **1** (1 eq) and dabco (0.5 eq).

R2: Slow evaporation of an ethylacetate solution containing compound **2** (1 eq) and dabco (0.5 eq).

R3: Mixing compound **3** (1 eq) and dabco (1 eq) in Ethylacetate/DMSO/DMF/ACN rapidly formed the obtained co-crystals. Complete evaporations of the solvent resulted into a homogeneous sample.

(b) Co-crystal structure details:

Table S1. Chalcogen bond parameters in crystal structure of **R1**, **R2** and **R3** at 300 K. Space group, Se•••N ChB distances involving σ-hole along (N≡)C−Se and (Ph)C−Se bond, and corresponding angles are indicated.

Co-crystal	Space group	Se•••N(dabco) (Å)	NC−Se•••N(dabco) (°)	Se•••N(CN) (Å)	(N≡/Ph)C−Se•••N(CN) (°)
**R1**	$P2_1/c$	2.723	173.9	-	-
**R2**	P1-	2.649	174.8	3.301	169.7
				3.261[a]	161.3[a]
**R3**	C2/c	2.852	173.8	3.290	171.2
				3.412[a]	170.6[a]
				3.224	164.5

[a]Related to Ph−Se•••N(CN) ChB where same Se atom is also bonded to DABCO through σ-hole along the N≡C−Se bond.

Table S2. Crystal data and structure refinement for **R1**.

**Temperature**	**100K**	**285K**
CCDC Codes	**2494001**	**2494004**
Empirical formula	$C_{13.33}H_{13.33}Cl_{1.33}N_{2.67}Se_{1.33}$	$C_{10}H_{10}ClN_2Se$
Formula weight	363.488	272.616
Temperature/K	100	285(3)
Crystal system	monoclinic	monoclinic
Space group	$P2_1/c$	$P2_1/c$
a/Å	6.1280(1)	6.2611(1)
b/Å	12.6645(2)	12.6637(2)
c/Å	13.7742(2)	14.0710(2)
α/°	90	90
β/°	90.378(1)	91.165(1)
γ/°	90	90
Volume/Å^3	1068.97(3)	1115.44(3)
Z	3	4
ρ_{calc}g/cm^3	1.694	1.623
μ/mm^{-1}	6.732	6.452
F(000)	539.5	539.5
Crystal size/mm^3	0.15 × 0.02 × 0.01	0.2 × 0.1 × 0.1
Radiation	Cu Kα (λ = 1.54184)	Cu Kα (λ = 1.54184)
2Θ range for data collection/°	9.48 to 153.22	9.4 to 153.38
Index ranges	$-7 \le h \le 7$, $-15 \le k \le 15$, $-12 \le l \le 17$	$-7 \le h \le 7$, $-15 \le k \le 15$, $-17 \le l \le 17$
Reflections collected	9904	20483
Independent reflections	2065 [R_{int} = 0.0496, R_{sigma} = 0.0382]	2273 [R_{int} = 0.0453, R_{sigma} = 0.0225]
Data/restraints/parameters	2065/18/191	2273/18/195
Goodness-of-fit on F^2	1.045	1.031
Final R indexes [I>=2σ (I)]	R_1 = 0.0292, wR_2 = 0.0685	R_1 = 0.0252, wR_2 = 0.0657
Final R indexes [all data]	R_1 = 0.0369, wR_2 = 0.0735	R_1 = 0.0335, wR_2 = 0.0713
Largest diff. peak/hole / e Å^{-3}	0.41/-0.46	0.26/-0.31

Table S3. Crystal data and structure refinement for **R2**.

**Temperature**	**100K**	**300K**
CCDC Code	**2494005**	**2494007**
Empirical formula	$C_{13}H_{14}N_3Se_2$	$C_{13}H_{14}N_3Se_2$
Formula weight	370.19	370.19
Temperature/K	99.9(2)	300.12(10)
Crystal system	triclinic	triclinic
Space group	P-1	P-1
a/Å	8.17755(16)	8.2661(2)
b/Å	8.59677(14)	8.7256(2)
c/Å	10.44428(13)	10.5621(2)
α/°	97.7282(12)	97.644(2)
β/°	96.8190(14)	97.059(2)
γ/°	90.9494(15)	90.356(2)
Volume/$Å^3$	722.03(2)	749.12(3)
Z	2	2
$\rho_{calc}g/cm^3$	1.703	1.641
μ/mm^{-1}	6.287	6.060
F(000)	362.0	362.0
Crystal size/mm^3	0.1 × 0.02 × 0.01	0.1 × 0.02 × 0.01
Radiation	Cu Kα (λ = 1.54184)	Cu Kα (λ = 1.54184)
2Θ range for data collection/°	8.608 to 152.872	8.514 to 153.434
Index ranges	$-9 \leq h \leq 10, -10 \leq k \leq 10, -13 \leq l \leq 12$	$-10 \leq h \leq 10, -10 \leq k \leq 10, -13 \leq l \leq 12$
Reflections collected	25059	27641
Independent reflections	2792 [R_{int} = 0.0379, R_{sigma} = 0.0189]	2955 [R_{int} = 0.0461, R_{sigma} = 0.0199]
Data/restraints/parameters	2792/12/229	2955/30/240
Goodness-of-fit on F^2	1.135	1.062
Final R indexes [I>=2σ (I)]	R_1 = 0.0235, wR_2 = 0.0632	R_1 = 0.0266, wR_2 = 0.0728
Final R indexes [all data]	R_1 = 0.0256, wR_2 = 0.0644	R_1 = 0.0317, wR_2 = 0.0765
Largest diff. peak/hole / e $Å^{-3}$	0.38/-0.51	0.30/-0.43

Table S4. Crystal data and structure refinement for **R3**.

**Temperature**	**100K**	**300K**
CCDC Codes	**2494008**	**2494009**
Empirical formula	$C_{28}H_{22.4}N_{4.8}Se_{3.2}$	$C_{17.5}H_{14}N_3Se_2$
Formula weight	678.78	424.24
Temperature/K	99.9(2)	300.19(10)
Crystal system	monoclinic	monoclinic
Space group	C2/c	C2/c
a/Å	18.5461(6)	18.8043(5)
b/Å	7.2361(2)	7.2606(2)
c/Å	26.3877(8)	26.5682(7)
α/°	90	90
β/°	110.350(4)	109.507(3)
γ/°	90	90
Volume/$Å^3$	3320.24(19)	3419.16(17)
Z	5	8
ρ_{calc}g/cm^3	1.697	1.648
μ/mm^{-1}	5.566	5.405
F(000)	1664.0	1664.0
Crystal size/mm^3	0.1 × 0.02 × 0.02	0.1 × 0.02 × 0.02
Radiation	Cu Kα (λ = 1.54184)	Cu Kα (λ = 1.54184)
2Θ range for data collection/°	7.146 to 152.844	7.06 to 153.548
Index ranges	$-22 \le h \le 22$, $-7 \le k \le 9$, $-31 \le l \le 32$	$-23 \le h \le 23$, $-8 \le k \le 9$, $-31 \le l \le 33$
Reflections collected	14951	30776
Independent reflections	3175 [R_{int} = 0.0422, R_{sigma} = 0.0335]	3492 [R_{int} = 0.0664, R_{sigma} = 0.0315]
Data/restraints/parameters	3175/12/280	3492/30/292
Goodness-of-fit on F^2	0.878	0.919
Final R indexes [I>=2σ (I)]	R_1 = 0.0455, wR_2 = 0.1458	R_1 = 0.0494, wR_2 = 0.1528
Final R indexes [all data]	R_1 = 0.0525, wR_2 = 0.1541	R_1 = 0.0650, wR_2 = 0.1707
Largest diff. peak/hole / e $Å^{-3}$	0.83/-0.62	1.05/-0.50

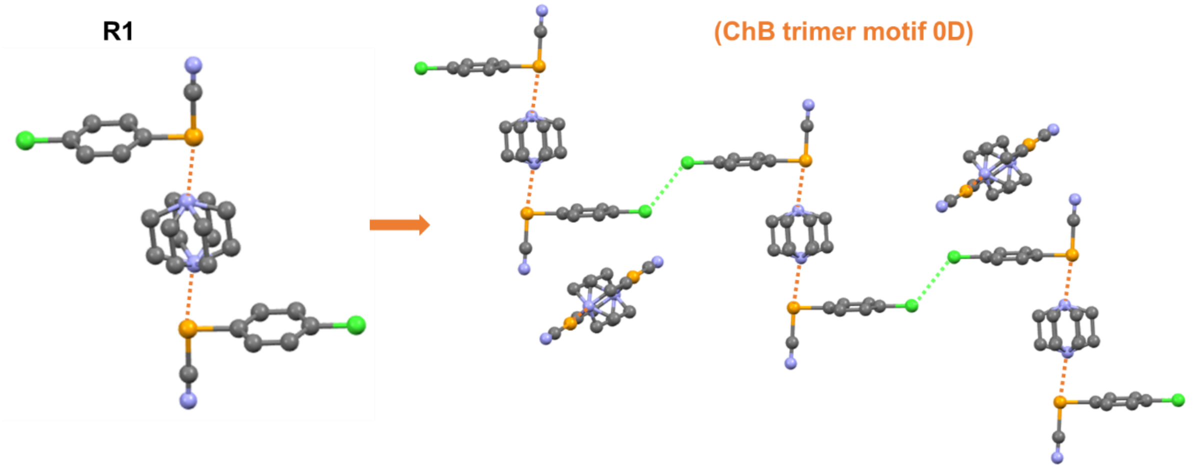

Figure S1. Partial view of the crystal structure of **R1** (ball and stick representation) showing Se•••N(dabco) contact pinning DABCO (left). Type I Cl•••Cl interactions (3.493 Å, 131.9°) are present between these isolated ChB bonded trimers. Hydrogens have been omitted for clarity. Color code: C, grey; N, sky blue; Se, orange.

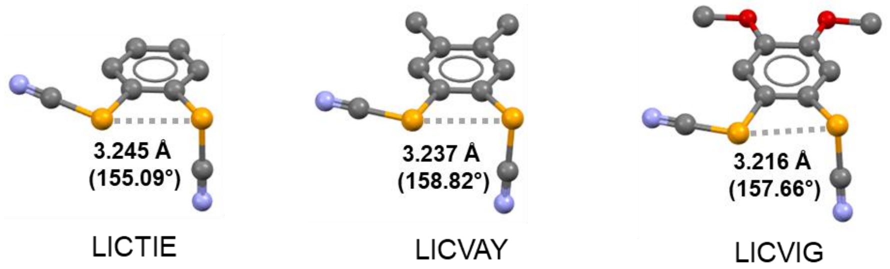

Figure S2. Solid-state molecular structures of known ortho substituted $Ph(SeCN)_2$ derivatives from crystal structure database (CSD) showing conformational locking via an intramolecular Se•••Se ChB (grey dotted lines) where σ-hole along N≡C−Se bond interacts attractively with lone pair of the second Se atom. The Se•••Se distance and (N≡)C−Se•••Se angles are indicated. Hydrogens have been omitted for clarity. Color code: C, grey; N, sky blue; O, red; Se, orange.

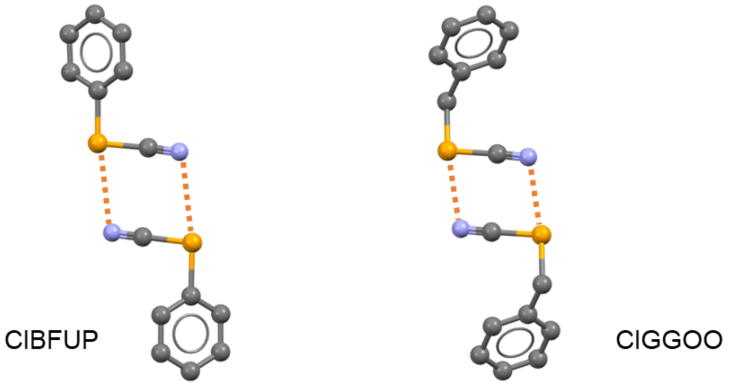

FIGURE S3. Partial view of the crystal structure of phenylselenocyanate and benzylselenocyanate (ball and stick representation) showing Se•••N(CN) contacts involved in recurrent antiparallel pairing of SeCN units. Hydrogens have been omitted for clarity. Color code: C, grey; N, sky blue; O, red; Se, orange.

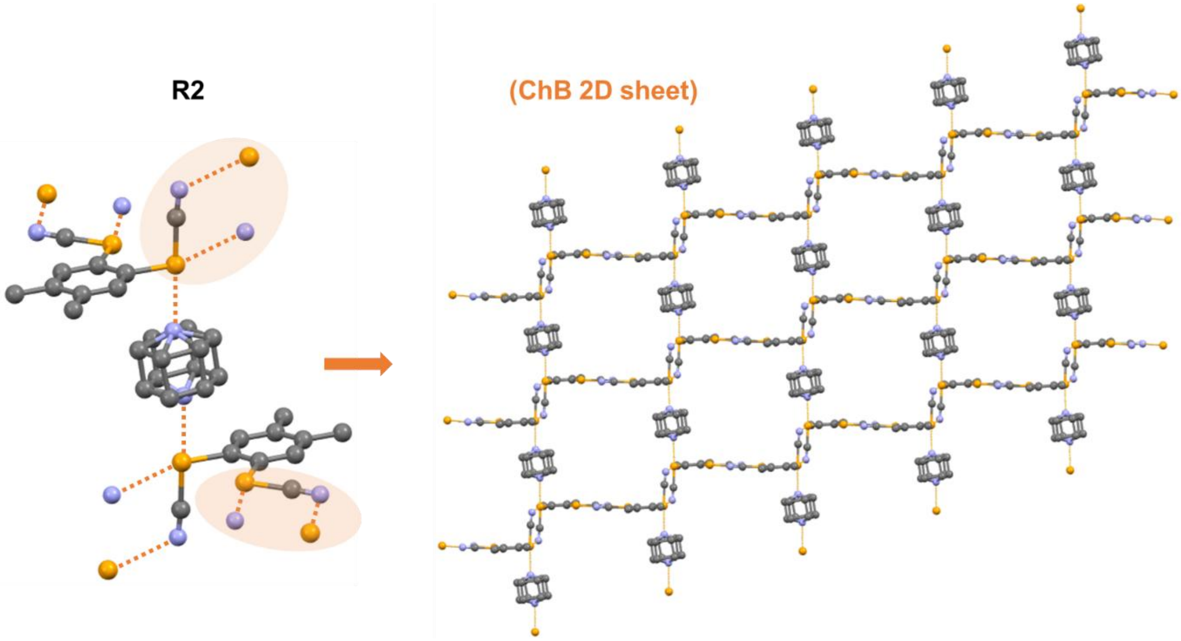

FIGURE S4. Partial view of the crystal structure of **R2** (ball and stick representation) showing Se•••N(dabco) and Se•••N(CN) contacts involved in pinning of DABCO and in antiparallel pairing of SeCN respectively (highlighted in circles). These ChBs together drive the assembly into a 2D sheet type network. Hydrogens have been omitted for clarity. Color code: C, grey; N, sky blue; O, red; Se, orange.

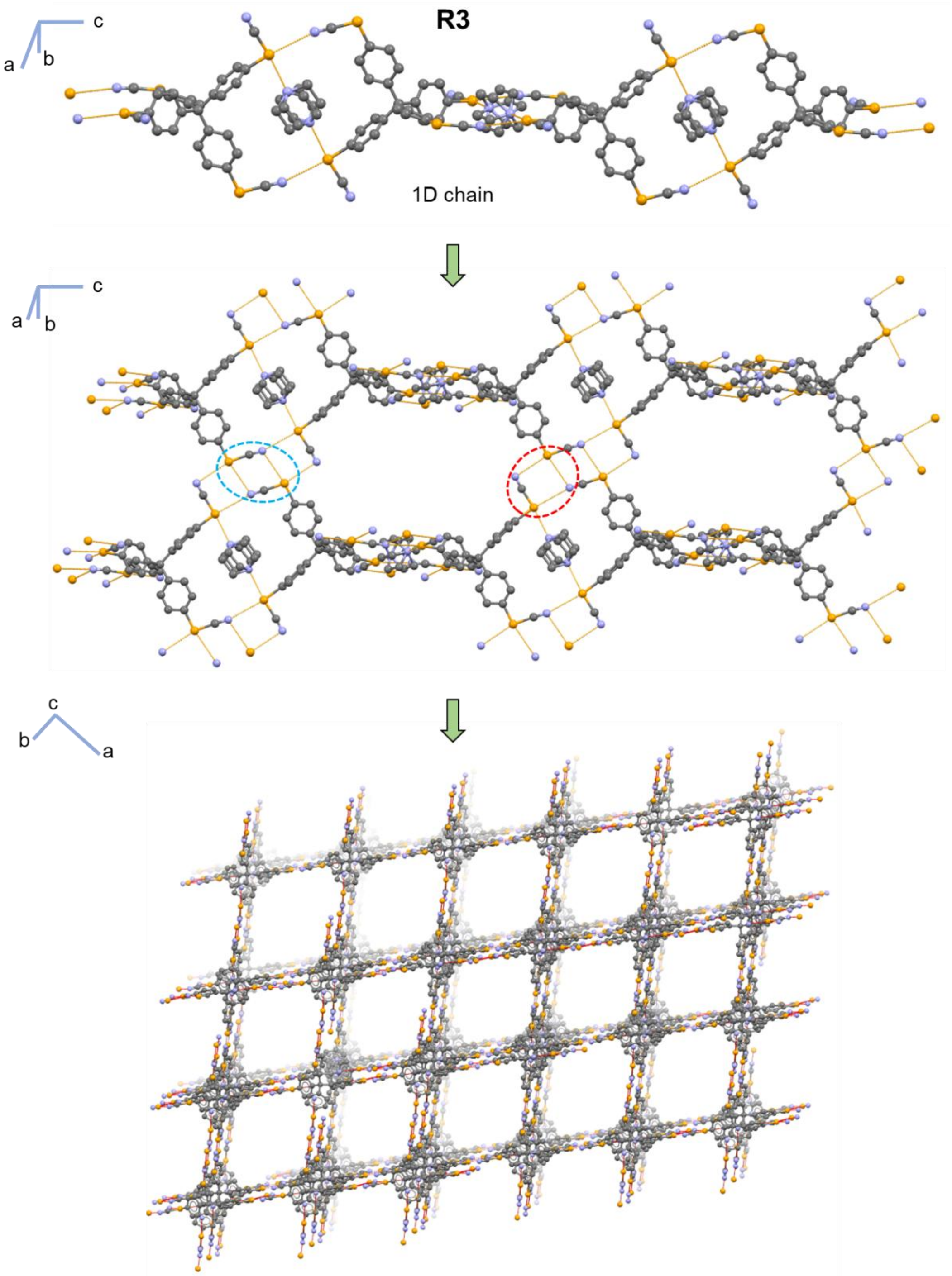

FIGURE S5. Partial view of the crystal structure of **R3** along a axis (ball and stick representation) showing Se•••N(dabco) and Se•••N(CN) contacts (orange dotted lines) pinning the DABCO inside a cavity and forming 1D chain motif (top). Antiparallel pairing of SeCN units is highlighted in blue dotted circle, as seen for **R2**. This motif forms Se•••N ChBs with another two SeCN units (red dotted circle) and extend ChB network in all three directions (middle). The view along c axis shows formation of a porous 3D ChB network (bottom). Hydrogens have been omitted for clarity. Color code: C, grey; N, sky blue; O, red; Se, orange.

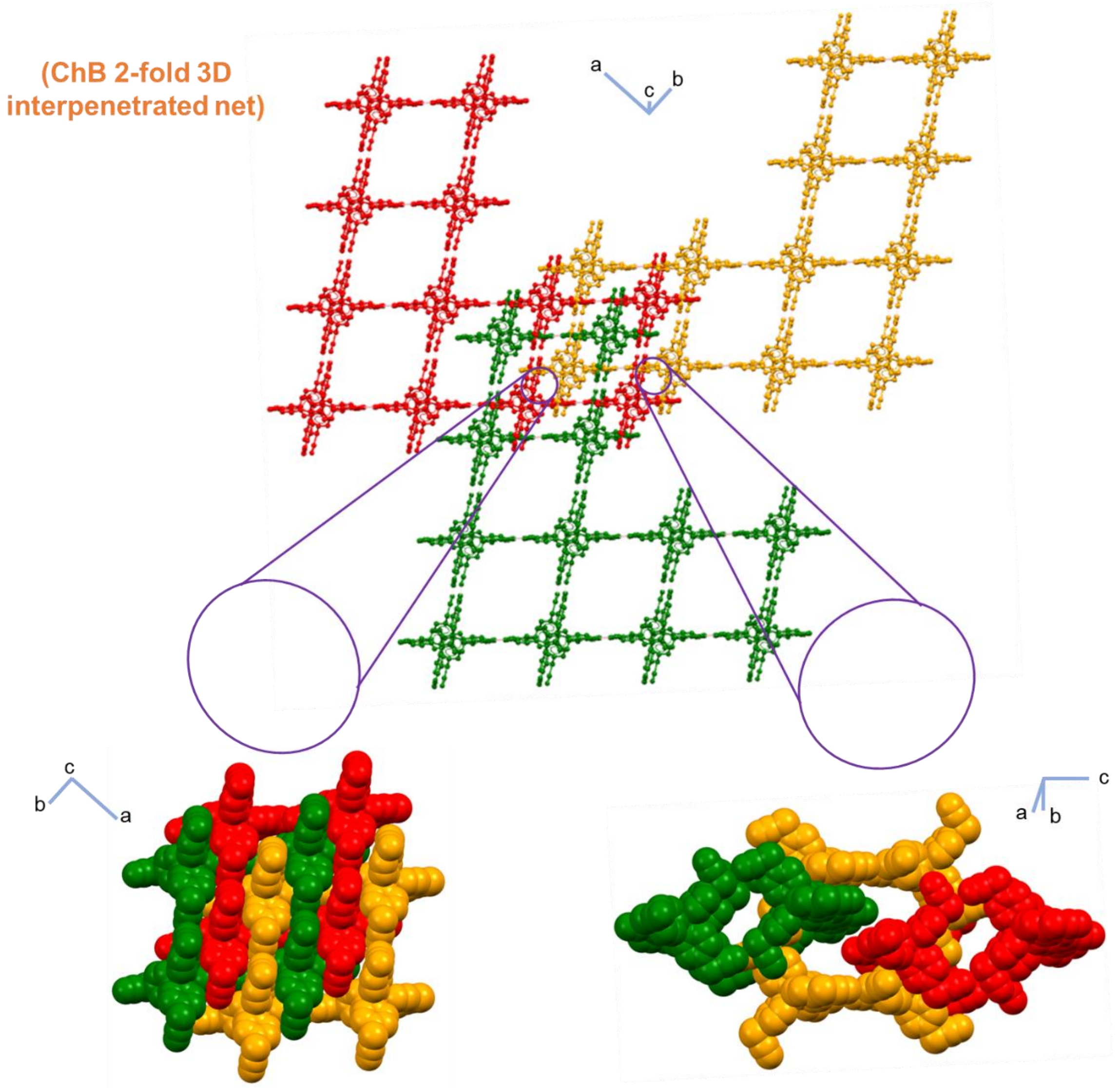

FIGURE S6. Partial view of the crystal structure of **R3** along c axis (ball and stick representation) showing relative positioning of three independent ChB 3D nets (orange, red and green) in the lattice (top). A 2-fold interpenetration of the chalcogen bonded 3D nets is evidenced below with space fill representation along two directions. The DABCO units and hydrogen atoms are not shown for clarity.

(c) Power X-ray data:

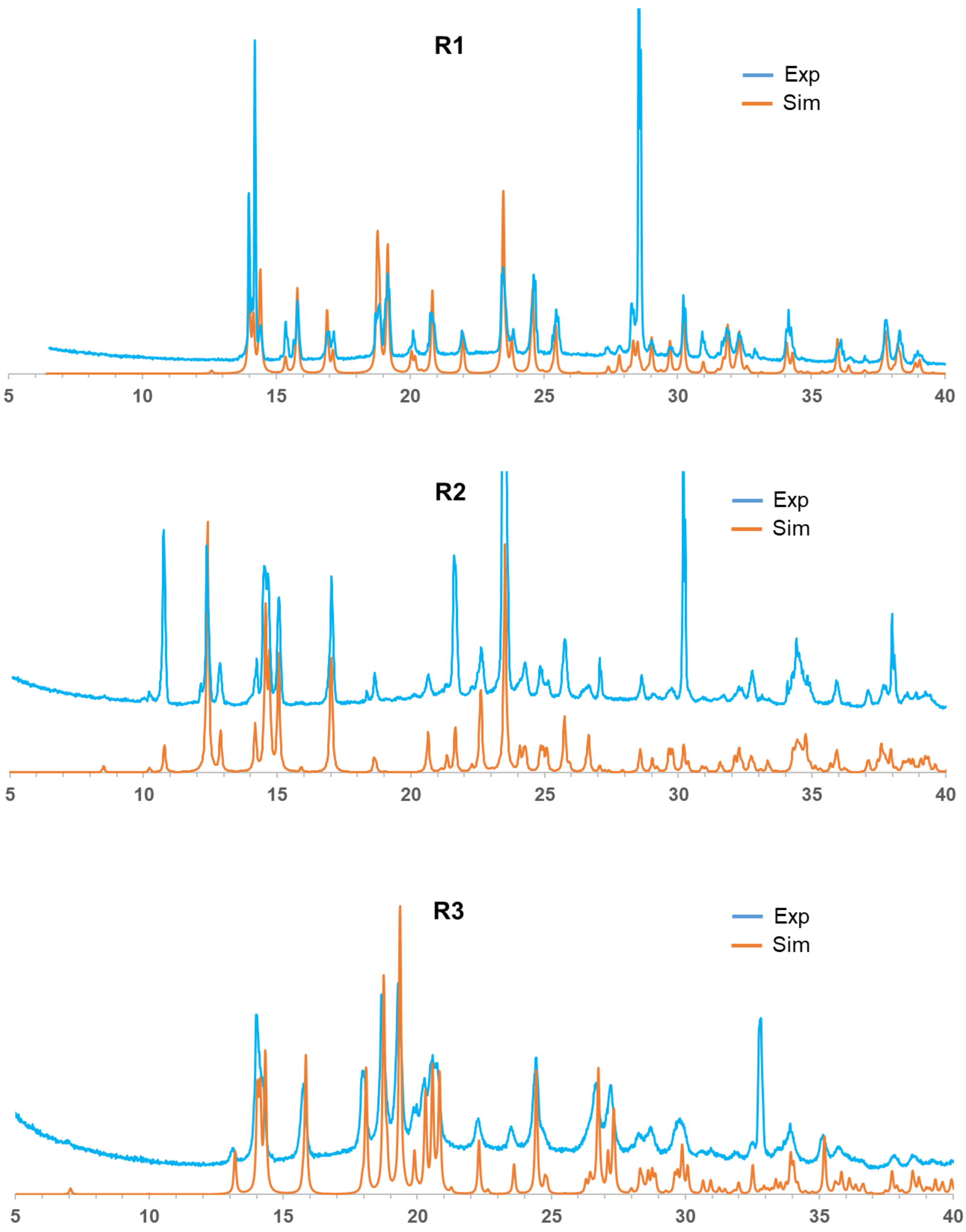

Figure S7. Powder x-ray pattens of co-crystals **R1** (top), **R2** (middle) and **R3** (bottom). The experimental data is shown in blue and the simulated patterns from single crystal x-ray structures are shown in orange. In each case, a consistent matching between the two patterns is observed, confirming the purity of obtained samples.

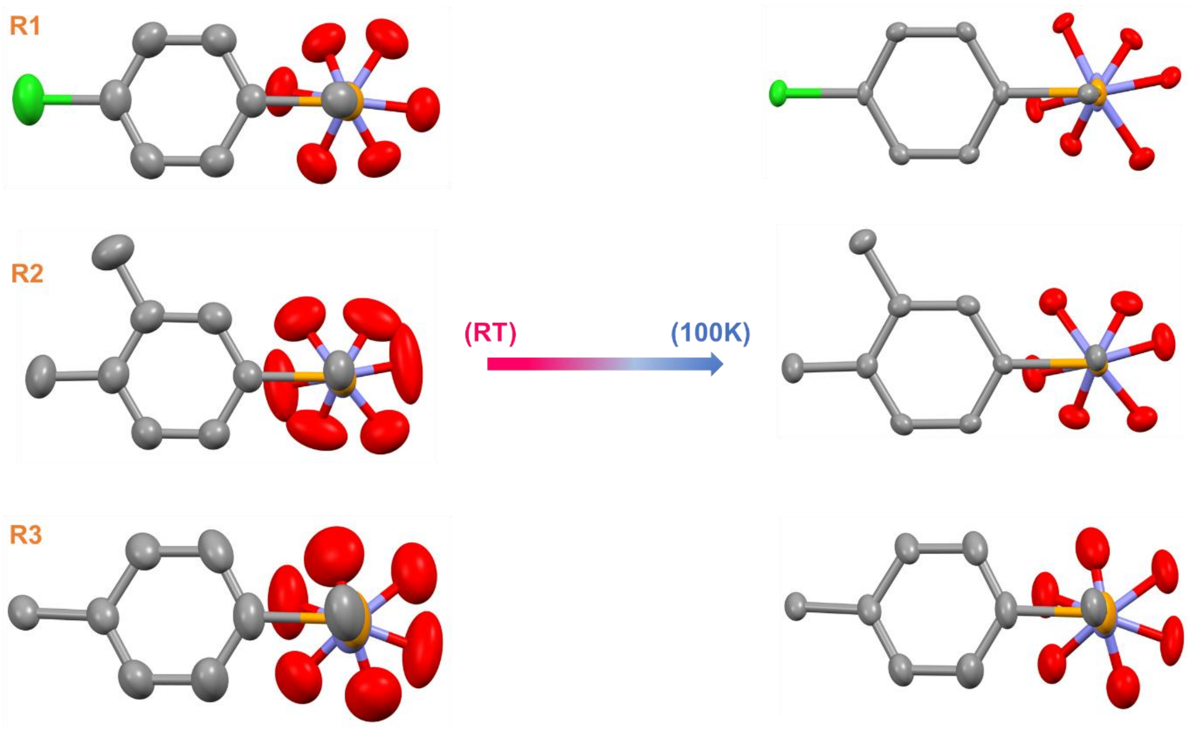

Figure S8. Partial view of the crystal structure of **R1**, **R2** and **R3** (ellipsoid representation) at RT and 100K, showing the differences observed in thermal ellipsoids of stator (C: grey) and rotator (C: red) parts. All hydrogens have been omitted for clarity.

(d) DSC analysis:

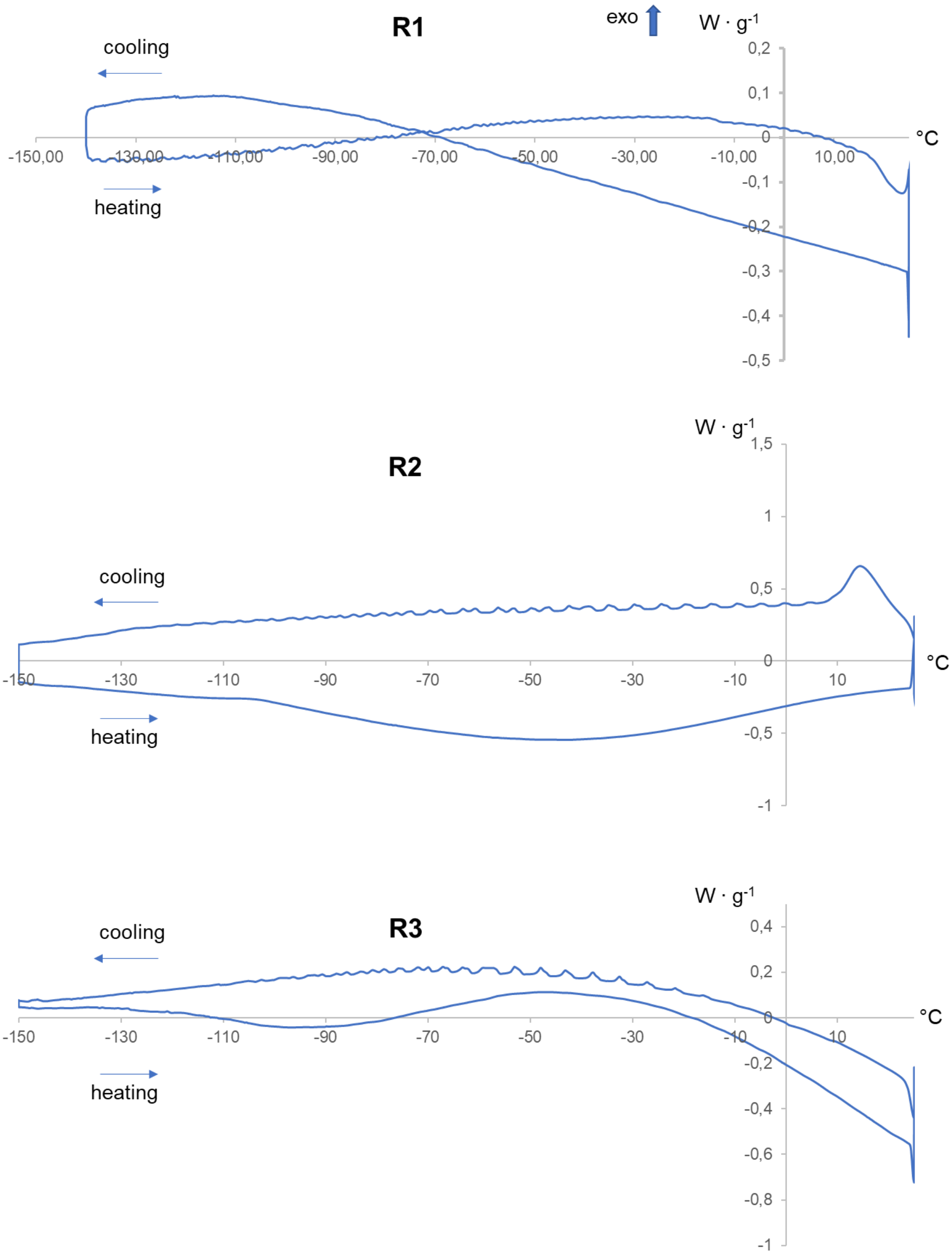

Figure S9. DSC profiles of co-crystals **R1** (top), **R2** (middle) and **R3** (bottom). Cool-heat cycle (-150 ºC to 25 ºC) showed no observable phase transitions. The lowest reachable temperature was -150 ºC.

Solid-state NMR studies:

(a) ^{13}C CPMAS ssNMR

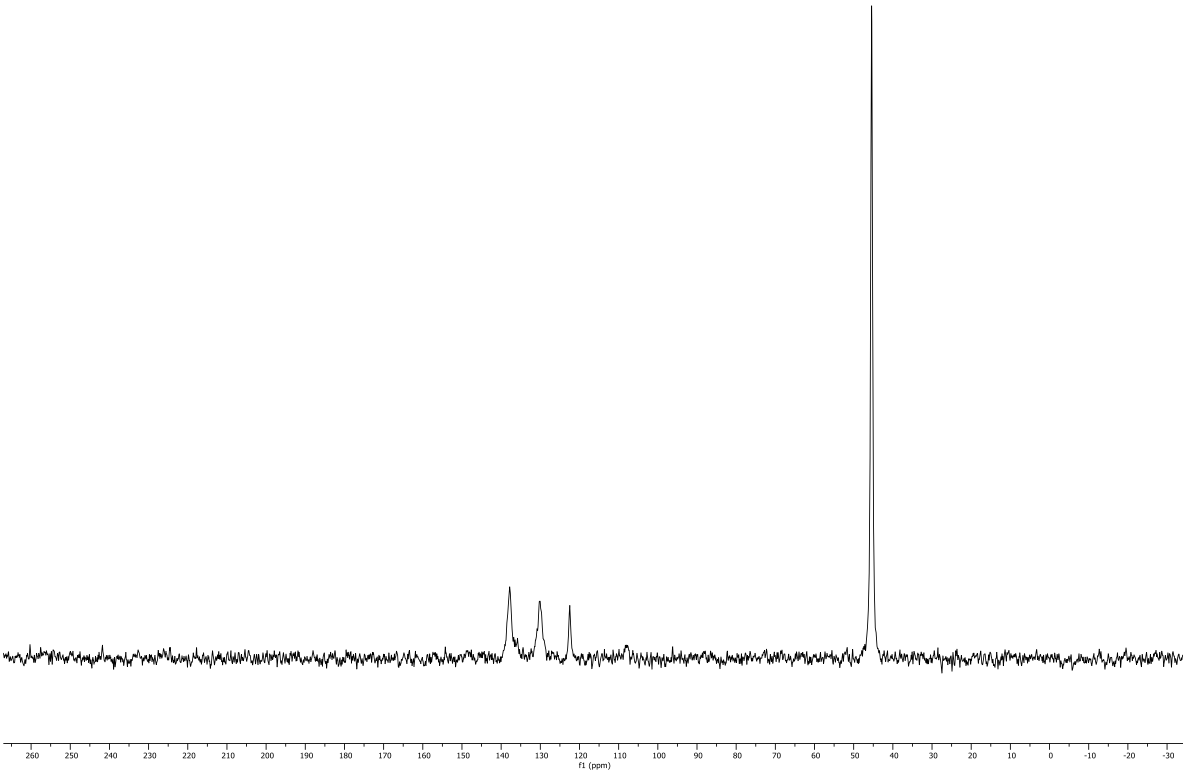

Figure S10. ^{13}C CPMAS ssNMR spectra of **R1**.

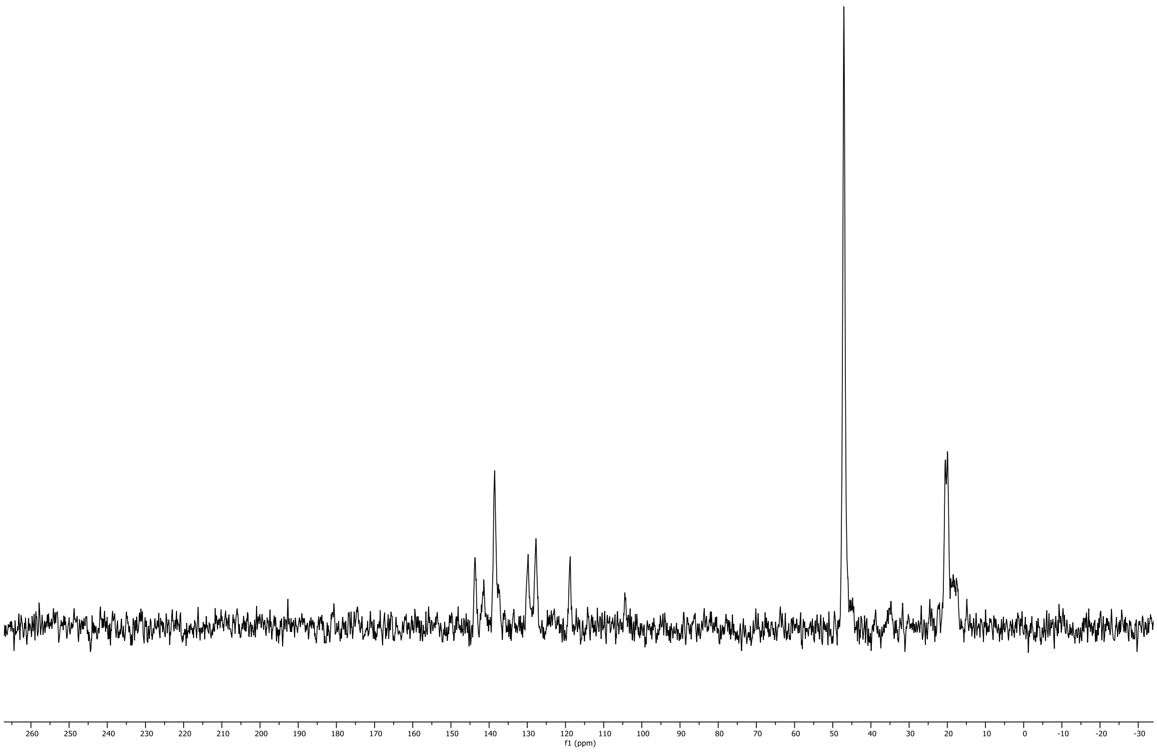

Figure S11. ^{13}C CPMAS ssNMR spectra of **R2**.

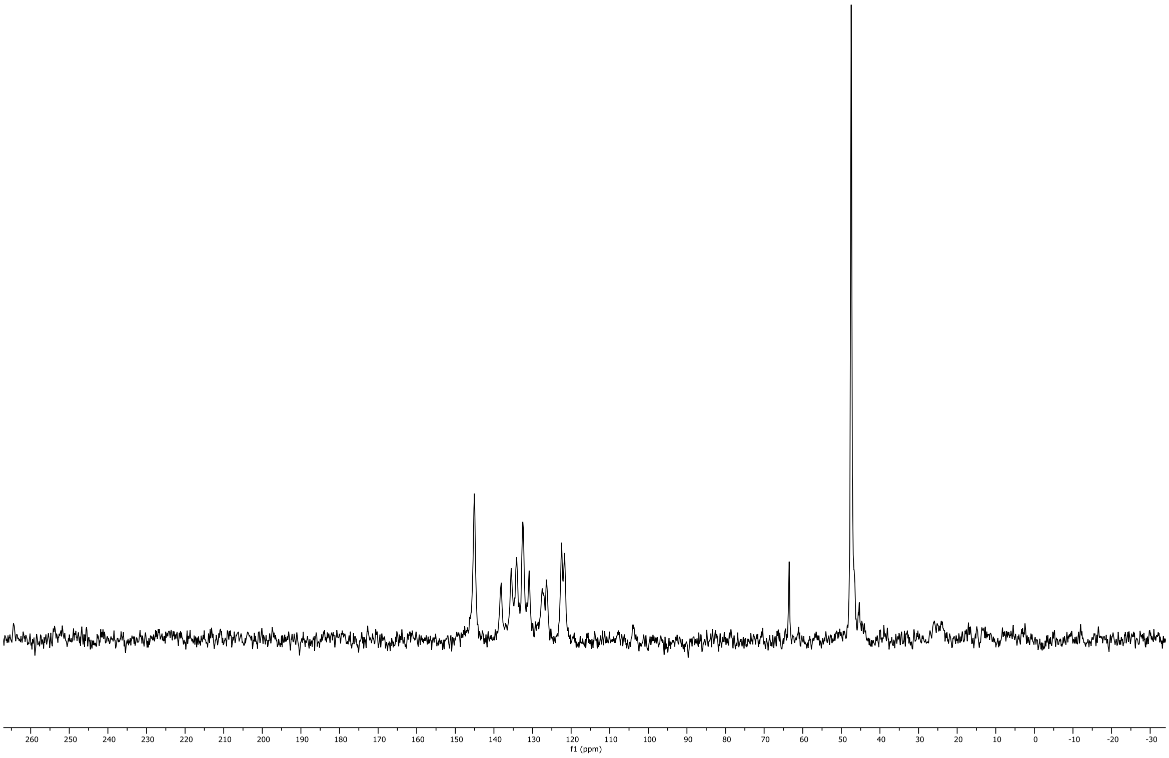

Figure S12. ^{13}C CPMAS ssNMR spectra of **R3**.

(b) T_1 spin-relaxation data

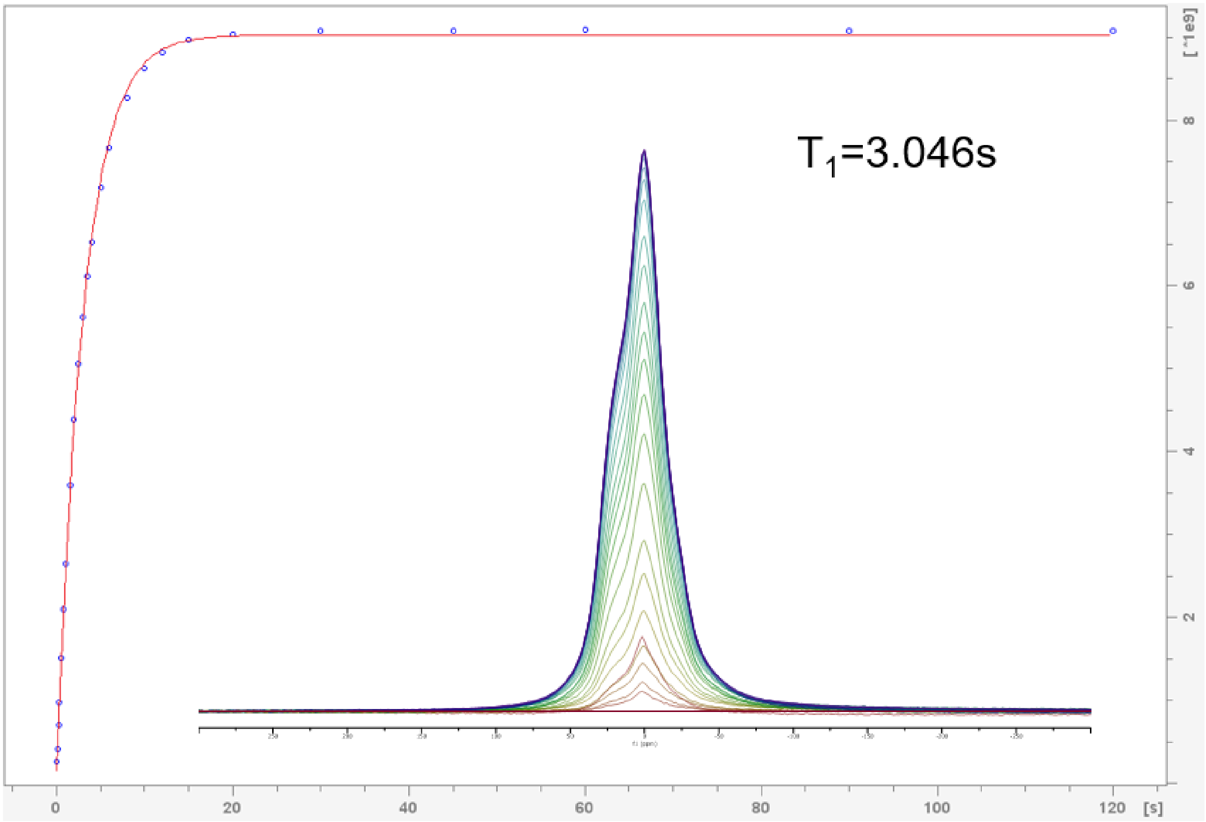

Figure S13. Representative T_1 relaxation curve of **R1** at 295K using *f* =600MHz.

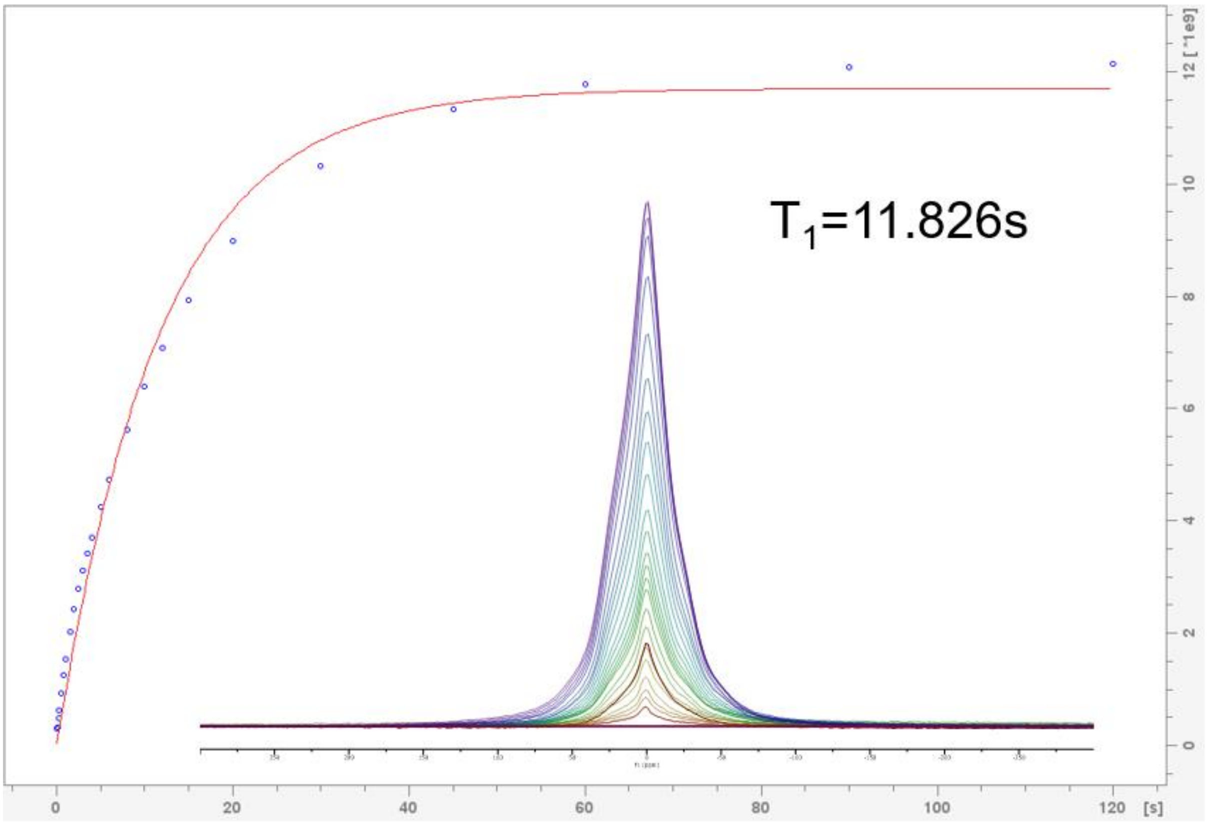

Figure S14. Representative T_1 relaxation curve of **R2** at 295K using f=600MHz.

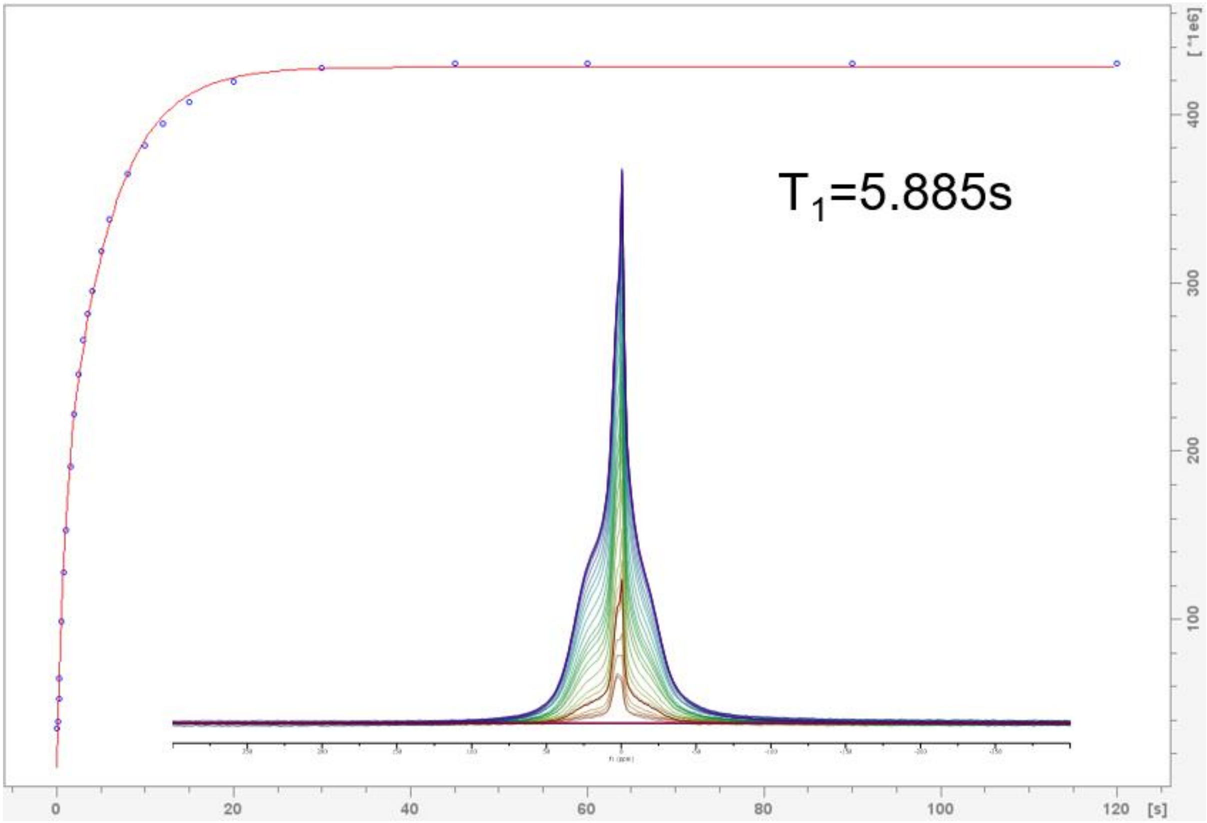

Figure S15. Representative T_1 relaxation curve of **R3** at 295K using f=600MHz.

(c) Kubo-Tomita fitting (red line) of experimentally measured spin-lattice relaxation rates:

The measurements were carried out on polycrystalline samples using a standard saturation recovery pulse sequence at f=600MHz for **R1** and **R3** and f=27MHz for **R2**, where f is the proton resonance frequency of the spectrometer and has the relation $\omega_0 = 2\pi f$. The full range of data points were measured at a different frequency for **R2** because the temperature limits of the spectrometer operating at f=600MHz were exceeded. Nonetheless, the energy parameters determined for **R2** at f=27MHz provide a good fit for a limited number of experimental data measured at f=600MHz (Figure S17).

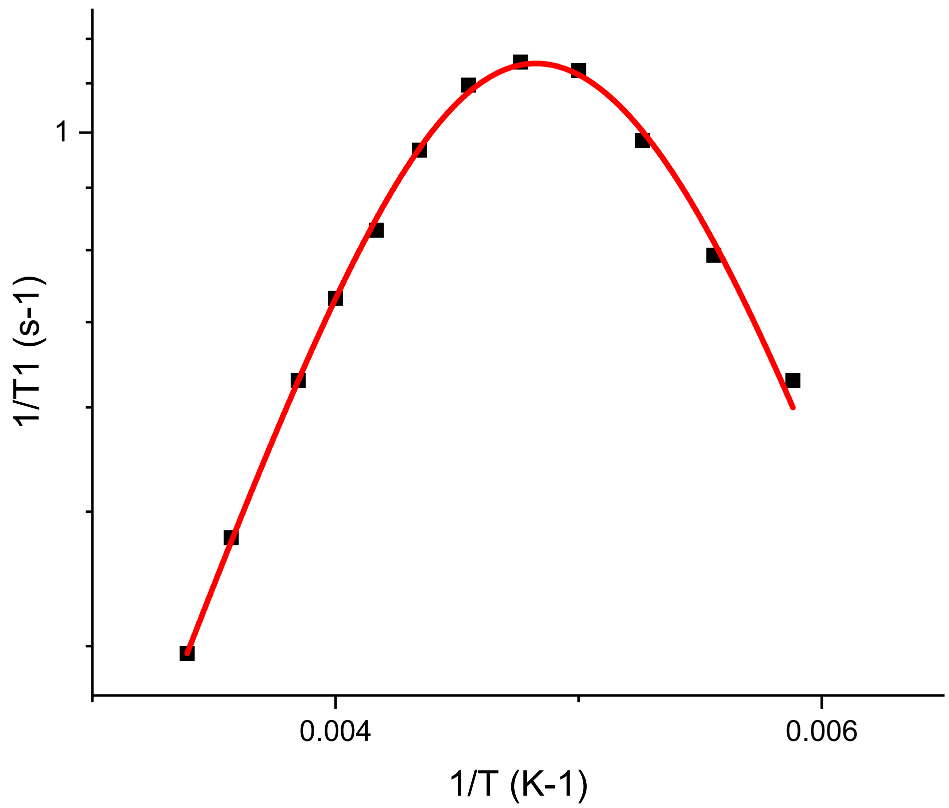

Figure S16. Kubo-Tomita fitting (—) of experimental T_1 values (■) for **R1** at f=600MHz.

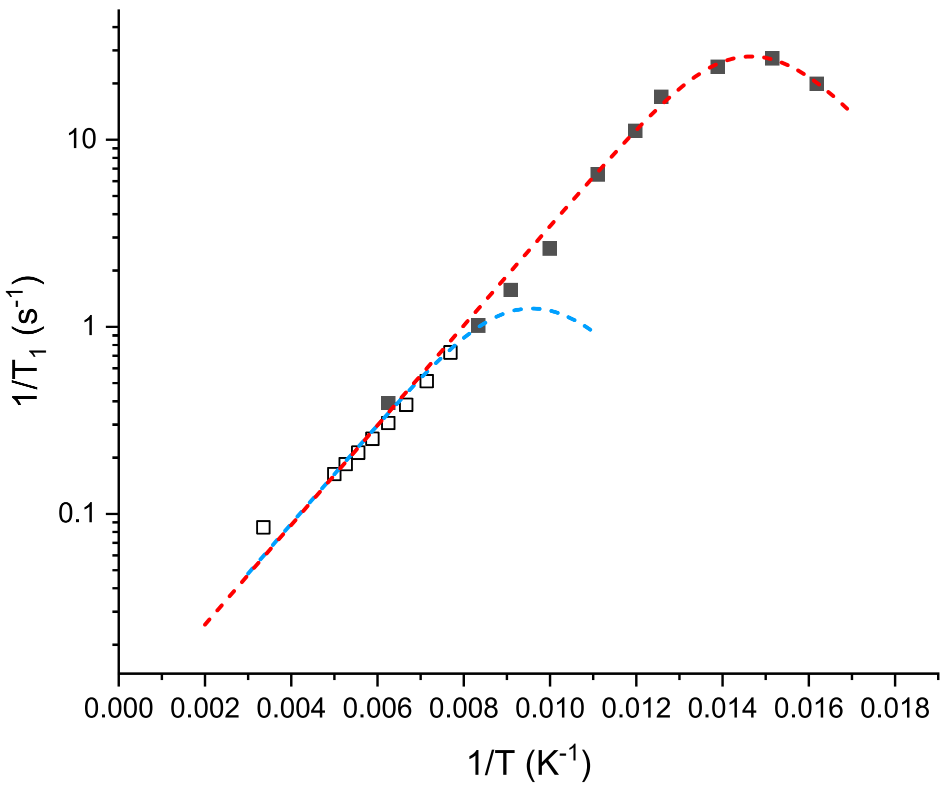

Figure S17. Kubo-Tomita fitting (dashed lines) of experimental T_1 values for **R2** at *f* =27MHz (■) and *f* =600MHz (□) using energy parameters in **Table 1** (main text).

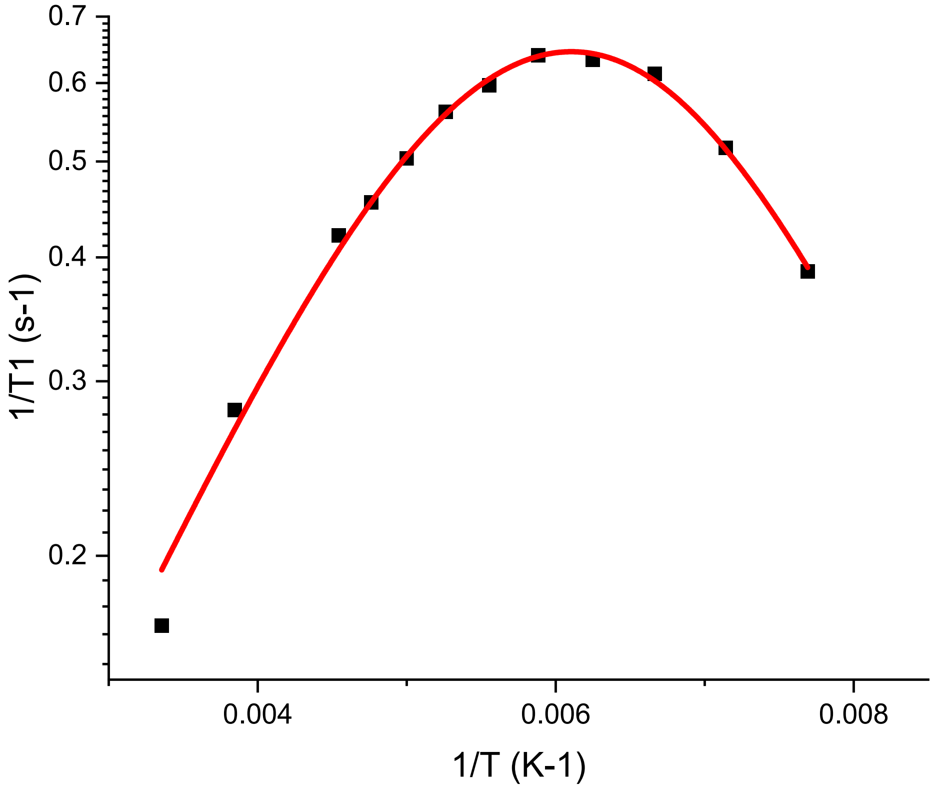

Figure S18. Kubo-Tomita fitting (—) of experimental T_1 values (■) for **R3** at *f* =600MHz.

Computational analysis of rotational dynamics:

(a) Packing coefficient calculations:

Calculations of the DABCO cavity volume were performed using Swiss-PdbViewer.[2] The cavity was defined by considering all surrounding molecules at distances ≤ ΣR_{vdW} (sum of the van der Waals radii) + 0.45 Å to establish the volume boundaries.

Table S5. Cavity and DABCO volumes (Å^3) and packing coefficients for **R1**-**R3** calculated with Swiss-PdbViewer.

Compound	Cavity (Å^3)	DABCO (Å^3)	Packing coefficient (%)
**R1**	200	112	56
**R2**	214	112	52
**R3**	225	112	50

(b) Rotational barrier calculations:

All calculations were performed at the PBE0-D4/def2-TZVP level of theory[3-5] without symmetry constraints using the TURBOMOLE 7.9 program.[6] Models were constructed using the X-ray structures at 100 K. Taking one DABCO molecule as a reference, we selected all surrounding molecules at ≤ ΣR_{vdW} + 0.3 Å. For **R3**, parts of the molecules distal to the central DABCO were truncated to maintain computational feasibility. These models are illustrated in Figure S19.

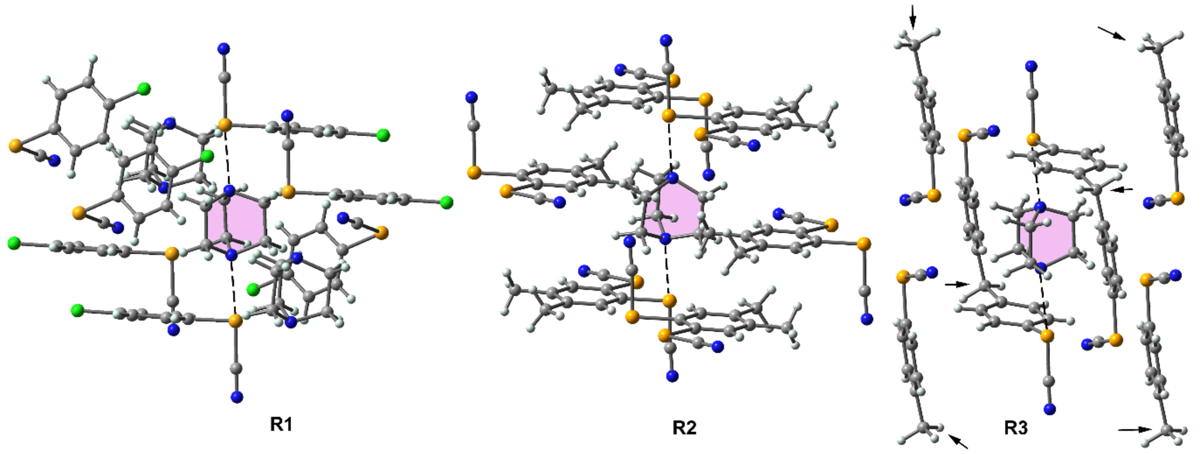

Figure S19. Initial geometries used for the scans. For **R3**, arrows indicate where the model was truncated by replacing phenyl-SeCN units with H-atoms.

Using models derived from the X-ray structures at 100 K, rotational energy scans were carried out every 15°. To maintain computational efficiency while representing the crystal environment, molecules within the sum of van der Waals radii plus 0.3 Å from the central DABCO were included and kept frozen, while the positions of the H-atoms were optimized. Because DABCO has C_3 symmetry, a 120° rotation was sufficient to describe the potential energy surface (see Figure 4 in MS). The initial orientation of the DABCO rotator, as determined from the X-ray structures, was assigned a rotation angle of 0° and represents the energy minimum for all three systems. To investigate the different contributions to the activation barrier, the Kitaura-Morokuma energy partition scheme[7] complemented with the D4 dispersion term was applied to the global minima and transition states. The potential energy scans reveal that the transition states, or energy maxima, are encountered at 30° and that the minimum at 60° is almost equivalent to the minima at 60° and 120° in line with the 50% occupation observed in the solid state structures. The relative energies from these scans are provided in Table S6.

Table S6. Relative energies (kcal·mol^{-1}) of rotamers from 0° to 120° at the PBE0-D4/def2-TZVP level of theory.

Rotamer	**R1**	**R2**	**R3**
0	0.00	0.00	0.00
15	1.21	0.48	0.86
30	2.19	0.99	1.50
45	1.11	0.46	0.74
60	0.25	0.09	0.01
75	1.11	0.46	0.74
90	2.19	0.99	1.50
105	1.21	0.48	0.86
120	0.00	0.00	0.00

References:

[1] Alfuth, J.; Jeannin, O.; Fourmigué, M. Topochemical, Single-Crystal-to-Single-Crystal [2+2] Photocycloadditions Driven by Chalcogen-Bonding Interactions. *Angew. Chem. Int. Ed.* **2022**, *61*, e202206249.

[2] Swiss-PdbViewer. https://spdbv.unil.ch/ accessed March 19, 2026

[3] Adamo, C.; Barone, V. Toward reliable density functional methods without adjustable parameters: The PBE0 model. *J. Chem. Phys*. **1999**, *110*, 6158-6170.

[4] Caldeweyher, E.; Ehlert, S.; Hansen, A.; Neugebauer, H.; Spicher, S.; Bannwarth, C.; Grimme, S. A generally applicable atomic-charge dependent London dispersion correction. *J. Chem. Phys*. **2019**, *150*, 154122.

[5] Weigend, F.; Ahlrichs, R. Balanced basis sets of split valence, triple zeta valence and quadruple zeta valence quality for H to Rn: Design and assessment of accuracy. *Phys. Chem. Chem. Phys*. **2005**, *7*, 3297-3305.

[6] Furche, F.; Ahlrichs, R.; Hattig, C.; Klopper, W.; Sierka, M.; Weigend, F. Turbomole. *WIREs Comput. Mol. Sci*. **2014**, *4*, 91-100.

[7] Kitaura, K.; Morokuma, K. A new energy decomposition scheme for molecular interactions within the Hartree-Fock approximation. *Int. J. Quantum Chem*. **1976**, *10*, 325-340.
